# Comprehensive Detoxification Mechanism Assessment of Red Imported Fire Ant (*Solenopsis invicta*) against Indoxacarb

**DOI:** 10.3390/molecules27030870

**Published:** 2022-01-27

**Authors:** Junaid Ali Siddiqui, Yuping Zhang, Yuanyuan Luo, Bamisope Steve Bamisile, Naveed Ur Rehman, Waqar Islam, Muhammad Qasim, Qiuying Jiang, Yijuan Xu

**Affiliations:** 1 Department of Entomology, South China Agricultural University, Guangzhou 510642, China or junaidali206@gmail.com (J.A.S.); bamisopebamisile@yahoo.com (B.S.B.); jqy@stu.scau.edu.cn (Q.J.); 2 Guangdong Provincial Key Laboratory of High Technology for Plant Protection, Plant Protection Research Institute, Guangdong Academy of Agricultural Sciences, Guangzhou 510640, China; 3 Institute for the Control of Agrochemicals, Ministry of Agriculture and Rural Affairs, Beijing 100125, China; luoyuanyuan@agri.gov.cn; 4 State Key Laboratory for Conservation and Utilization of Subtropical Agro-Bioresources, Guangdong, Provincial Key Laboratory of Plant Molecular Breeding, South China Agricultural University, Guangzhou 510642, China; naveed@scau.edu.cn; 5 State Key Laboratory of Desert and Oasis Ecology, Xinjiang Institute of Ecology and Geography, Chinese Academy of Sciences, Urumqi 830011, China; Ddoapsial@yahoo.com; 6 University of Chinese Academy of Sciences, Beijing 100049, China; 7 Department of Agriculture and Forestry, Kohsar University Murree, Murree 47150, Pakistan; mqasim@kum.edu.cn

**Keywords:** drug resistance, toxicology, enzyme activity, carboxylesterase, glutathione S-transferases, xenobiotic stress

## Abstract

The red imported fire ant *(Solenopsis invicta)* is one of the deadliest invasive ant species that threatens the world by disrupting biodiversity, important functions within a natural ecosystem, and community structure. They are responsible for huge economic losses in the infested countries every year. Synthetic insecticides, especially indoxacarb, have been broadly used to control *S. invicta* for many years. However, the biochemical response of *S. invicta* to indoxacarb remains largely undiscovered. Here, we used the sublethal doses of indoxacarb on the *S. invicta* collected from the eight different cities of Southern China. The alteration in the transcriptome profile of *S. invicta* following sublethal dosages of indoxacarb was characterized using high-throughput RNA-seq technology. We created 2 libraries, with 50.93 million and 47.44 million clean reads for indoxacarb treatment and control, respectively. A total of 2018 unigenes were regulated after insecticide treatment. Results indicated that a total of 158 differentially expressed genes (DEGs) were identified in the indoxacarb-treated group, of which 100 were significantly upregulated and 58 were downregulated, mostly belonging to the detoxification enzymes, such as AChE, CarE, and GSTs. Furthermore, results showed that most of these DEGs were found in several KEGG pathways, including steroid biosynthesis, other drug metabolizing enzymes, glycerolipid metabolism, chemical carcinogenesis, drug-metabolizing cytochrome P450, glutathione metabolism, glycerophospholipid metabolism, glycolysis/gluconeogenesis, and metabolism of xenobiotics. Together, these findings indicated that indoxacarb causes significant alteration in the transcriptome profile and signaling pathways of *S. invicta*, providing a foundation for further molecular inquiry.

## 1. Introduction

Social insects, mostly ants, are one of the most devastating invaders [1]. Features that define them as highly successful intruders include super colony assemblage, great replicability, and aggressive monopolization of natural systems to overcome indigenous species [2]. Among the invasive species that pose the most severe danger to the ecosystems is the red imported fire ant (RIFA) (*Solenopsis invicta*), which has spread to every continent of the planet [1,3]. *Solenopsis invicta* is a South American native, which has been introduced worldwide, including in China [4]. This invasive species was initially discovered in Taiwan in 2003 [5] and then later in Guangdong Province in 2004 [6]. Most recently, *S. invicta* has been reported in more than 280 countries throughout South China [7,8,9]. Invasive species can have a direct impact on native biodiversity, such as herbivores preying on local plants [10], predators or parasitoids attacking native prey or hosts [11,12], and through hybridizing with native species [13]. Furthermore, cascading effects or various processes can indirectly affect native species and ecosystems. For example, they might spread disease to native species and ecosystems, compete for food and space, or share natural enemies with native species [1]. The rapid spread of invasive ants from one location to another by humans is made possible due to their generalist tendencies, tiny size, and frequent encounters with dwellings or ecological damage [14], allowing for their establishment and subsequent dissemination [15]. That is why the populations of *S. invicta* are abundantly found around the coastal cities of Guangdong province, China.

Synthetic pesticides are frequently used to control *S. invicta* [16]. Chemical pesticides are heavily utilized to manage various invasive ants in the current era. Despite their effectiveness, numerous questions have been raised about the side effects of these synthetic insecticides, such as developing resistance in target insects, pollution of the environment, and negative influence on human health [17]. In China, several synthetic chemical products have been registered to manage *S. invicta* [4]. Recently, indoxacarb has been used widely against the red imported fire ant (RIFA) [4]. Indoxacarb is a new foliar chemical with a broad-spectrum application, not only for the field management of lepidopterans. Still, it has also been confirmed to exhibit high effectiveness against various key insects, including ants, cockroaches, leafhoppers, and aphids [18]. The primary mode of action of indoxacarb is to block the sodium channel in the nerve cells of the target pest via N-decarbomethoxyllated metabolite, which potentially causes disintegration of the midgut [19]. The current study evaluated the effects of indoxacarb at sublethal concentrations (LC_10_ and LC_30_). Sublethal effects are biological, demographic, physiological, or behavioral changes in individuals or communities exposed to a toxicant at a lethal or sublethal concentration.

A sublethal concentration causes no apparent or less mortality [20]. Sublethal concentrations of insecticides are generally considered below the median lethal concentration (LD_50_/LC_50_) [21,22,23,24]. In contrast to the majority of evolutionary events, resistance has significant practical and economic implications. There has been a considerable increase in the number of resistant species as well [1], but the severity and scope of specific resistance problems have rapidly grown. However, the excessive use of insecticides can cause resistance in insects. A similar outcome might also occur in the case of RIFA [25] because indoxacarb has been used against fire ants for many years. The evaluations of resistance status are critical in effectively managing an insect pest.

The detoxification process is an essential and sophisticated system that detoxifies or eliminates many toxic substances, such as insecticides [26,27]. Detoxification enzymes are naturally involved in various biological processes, acting on the target location to neutralize various poisons found in the insect body [28]. According to the earlier studies, the biochemical characterization of insect resistance to an insecticide is related to target site insensitivity and pesticide detoxification by metabolic enzymes (AChE, CarE, and GSTs) [29,30,31,32]. These enzymes play an essential role in detoxifying xenobiotics [33]. During the pesticide detoxification processes, these enzymes serve as biological markers to analyze the degree of resistance, tolerance, or susceptibility present in the bodies of organisms [22]. Acetylcholinesterase (ACHE) is an essential enzyme for catalyzing neurotransmitter hydrolysis. Acetylcholine is found in the nervous system of different organisms [29,34]. Carboxylesterase (CarE) is a significant detoxifying enzyme that has a role in pesticide resistance [35,36]. It has been discovered that *Rhopalosiphum padi* exhibits CarE-mediated pesticide resistance [37]. Glutathione S-transferases (GST) are the most typically implicated enzymes in detoxifying xenobiotics [38,39]. Insect resistance to pesticides is primarily caused by the amplified activity of these enzymes or the production of new isoforms [40]. Zhang et al. (2016) indicated that the detoxification enzymes (cytochrome P450 genes) were found in the detoxification of fipronil and showed a 36.4-fold increase in the resistance in RIFA. Another study showed that the cytochrome P450 enzymes played a vital role in the detoxifications of the fluralaner [41]. The job of detoxifying enzymes, on the other hand, is the protection of insects from the harmful effects of insecticides on their bodies. These enzymes can also help break down hormones, pheromones, and other bioactive compounds. Therefore, variations in the activities of detoxication enzymes indicate insect resistance to insecticides and their adaptability to their host plant, their metamorphosis, and growth [42].

Moreover, these enzymes contribute to chemical balance, which is critical for various physiological processes in insects. Pesticides have been shown to disrupt the enzymatic equilibrium required for the performance of various physiological functions [43]. Two mechanisms primarily cause pesticide resistance: target site sensitivity and metabolic resistance caused by higher levels of insecticide detoxifying enzymes [44]. In several insect species, biochemical experiments have shown that insensitive esterase causes resistance to carbamates and organophosphates [45,46]. To determine the mechanism of sublethal effects of indoxacarb on *S. invicta* workers, the activities of acetylcholinesterase (AChE), carboxylesterase (CarE), and glutathione S-transferases (GST) along with the transcriptional changes were examined in the surviving fire ant workers from different populations after treatment by indoxacarb.

## 2. Results

### 2.1. Toxicity Bioassay

The toxicity of indoxacarb was investigated on *S. invicta* workers (Table 1). Results revealed that the computed LC_50_ of indoxacarb was highest for the colony collected from Guangzhou-2 (GZ-2) 0.02 (%), followed by the populations collected from Guangzhou-1 (GZ-1) 0.018 (%), Heyuan (HY) 0.011 (%), Huizhou (HZ) 0.014 (%), Shenzhen (SZ) 0.011 (%), Dongguan (DG) 1.18 × 10^−7^ (%), Jiangmen (JM) 9.37 × 10^−8^ (%), Zhongshan (ZS) 6.43 × 10^−8^ (%), and Zhuhai (ZH) 5.67 × 10^−8^ (%). According to the resistance ratio, it was revealed that *S. invicta* populations from GZ-2, were more resistant against indoxacarb compared with other populations, while ZH populations were the most susceptible to indoxacarb. The analysis of the resistance ratio also complements the toxicity bioassay results and elaborates that the resistance of the GZ-2 population was 2.12-fold higher than the most susceptible populations from ZH.

### 2.2. Enzyme Activity

**Figure 1:**
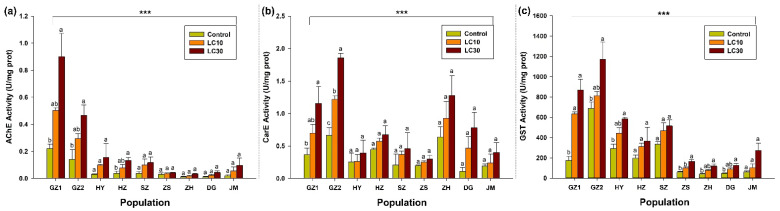
Enzyme activity of (**a**) AChE, (**b**) CarE, and (**c**) GST enzymes in response of indoxacarb in the population from GZ, ZS, ZH, DG, and JM at the sublethal concentration of indoxacarb. The bars represent the average of three replicates. Error bars show the standard error of the mean. The control and two treatments (LC_10_ and LC_30_) were compared by using the Tukey HSD all-pairwise comparison test. Bars with the same letters from a specific population showed non-significant differences and asterisks indicating the significant difference in enzyme activity between populations (*** *p* < 0.01, Tukey test).

Three detoxification enzymes, AChE, CarE, and GST, were used to assess the enzymes’ activity in different populations exposed to the sublethal concentrations of indoxacarb. The results indicated that the activity of AChE was significantly amplified across all collected populations in response to an increase in sublethal concentrations of indoxacarb. Moreover, the results indicated that the activity of AChE was significantly higher in the populations collected from GZ-1 compared with other populations (F_2,6_ = 11.3, *p* < 0.009), while AChE was less in the population collected from ZH (F_2,6_ = 4.8, *p* < 0.05) (Figure 1a). According to the results, the activity of CarE was significantly higher in the population collected from GZ-2 (F_2,6_ = 50.7, *p* < 0.002) as compared with the activity observed in populations of other regions, followed by activity observed in ZH, GZ-1, HZ, DG, HY, SZ, and JM, while CarE was less in the population collected from ZS (F_2,6_ = 1.77, *p* < 0.25) (Figure 1b). Similarly, with regard to the activity of GST, the highest activity was recorded in GZ-2, which was significantly different from the GST activity observed among other collected populations—GZ-1, HY, SZ, HZ, JM, and ZS (F _2,6_ = 1.77, *p* < 0.25; Figure 1b)—while CarE was less in the population collected from ZS (F_2,6_ = 19.1, *p* < 0.0025) and ZH (F_2,6_ = 9.22, *p* < 0.015) (Figure 1c).

### 2.3. Transcriptional Analysis of Indoxacarb Treated S. invicta

The sequencing libraries from the results of 6 samples of the most resistant populations (3 referred to control and 3 referred to LC_30_ treatments) of *S. invicta* after 24 h showed that total raw reads were 47.55 million and 51.08 million, and clean reads were 47.44 million and 50.93 million in control and treated groups, respectively (Table S5). After mapping to the reference genome ((http://ftp.ncbi.nlm.nih.gov/genomes/all/GCF/000/789/215/GCF_000789215.1_ASM78921v2) accessed on 21 January 2022) and the quality control procedures, the number of raw reads obtained from the 6 sequencing libraries ranged from 65711601 to 87310557, and the values of clean reads ranged from 652748709 to 8646724452 (Table S6). Unigenes from 6 samples had a Q30 value greater than 93.26% and a GC percentage ranging from 37.45 to 44.72% (Table S6).

### 2.4. KEGG Analysis

**Figure 2:**
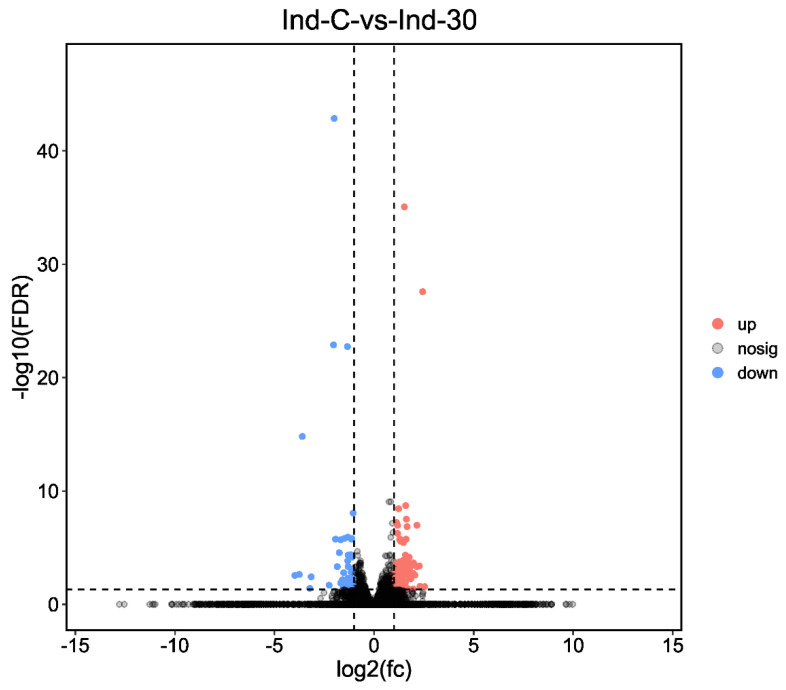
Differentially expressed unigenes after 24 h of exposure in indoxacarb-treated (Ind-30) and control (Ind-C) group. The fold change in gene expression is represented on the X-axis. The dotted x-axis indicates the relevance of gene expression statistically. *p*-value less than 0.05 is shown on the y-axis by −log10 (FDR). Scattered dots represent different genes. The grey dots represented no significant regulation, orange dots showed significantly upregulated genes, and blue dots showed significantly downregulated genes.

DEGs were compared between control and indoxacarb-treated *S. invicta,* the examination of DEGs (indoxacarb exposure for 24 h) discovered that a total of 2018 unigenes were found to be regulated due to pesticide exposure, from which 100 unigenes were significantly upregulated and 58 unigenes were significantly downregulated when indoxacarb was exposed to *S. invicta* workers (Log2FC > 2, *p* < 0.05) (Figure 2).

### 2.5. KEGG Analysis of DEGs

**Figure 3:**
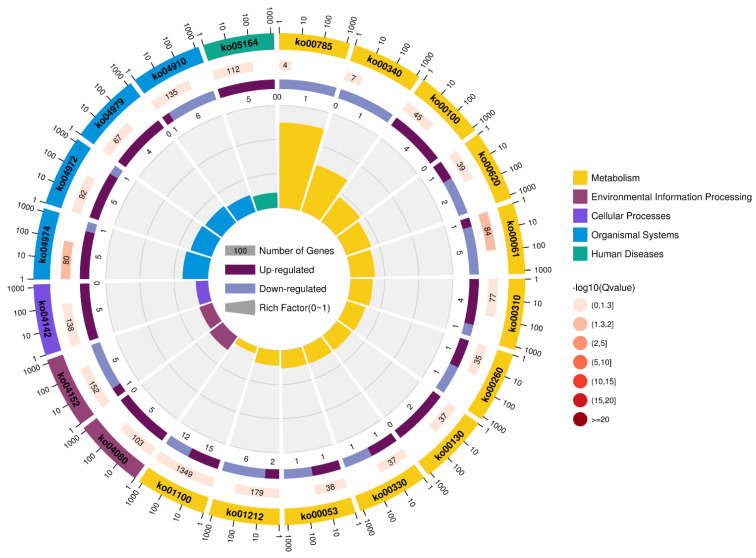
The most enhanced Kyoto Encyclopedia of Genes and Genomes (KEGG) pathways of *S. invicta* after indoxacarb exposure at sublethal concentrations (after 24 h). The significance of pathways is indicated by the *q*-value (color bar), the rich factor (X-axis), and the circle’s diameter, which indicates the number of genes regulated.

The investigation of DEGs via KEGG (indoxacarb exposure for 24 h) displayed that metabolic pathways, fatty acid metabolism, and insulin signaling pathways were the pathways comprising the maximum highly regulated unigenes 27 DEGs (*p* = 0.0169), 8 DEGs (*p* = 0.003), and 7 DEGs (*p* = 0.002419), respectively. Moreover, the metabolic pathways (15 DEGs), protein digestion and absorption (5 DEGs; *p* = 0.0007549), and pancreatic secretion (5 DEGs; *p* = 0.001574) comprised the maximum upregulated DEGs. On the other hand, metabolic pathways (12 DEGs), fatty acid metabolism and insulin signaling pathways (6 DEGs), and fatty acid biosynthesis (5 DEGs; *p* = 0.0009782) comprised the maximum downregulated DEGs (Figure 2 and Figure 3, and Table S8).

### 2.6. Highly Regulated GO Terms

**Figure 4:**
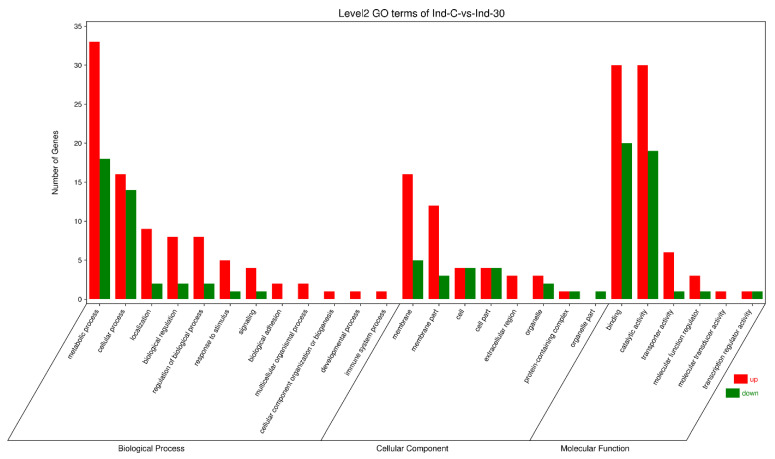
DEGs of *S. invicta* enriched in gene ontology (GO) following sublethal indoxacarb exposure (after 24 h). The x-axis denotes biological process (BP), cellular component (CC), and molecular function sub-Go terms (MF). The y-axis shows the DEGs participating in each term.

The gene ontology analysis of the control and indoxacarb-treated *S. invicta* samples revealed that 76 GO terms were significantly influenced, with 50 DEGs in catalytic activity, 24 DEGs in hydrolase activity, and 22 DEGs in membrane being among the most significantly regulated terms. The most upregulated GO terms were the catalytic activity with 30 DEGs, hydrolase activity with 18 DEGs, and membrane with 16 DEGs. In the catalytic activity, 20 DEGs, hydrolase activity and membrane with 6 DEGs, and catalytic activity, acting on a protein, with 5 DEGs, were downregulated GO terms. The GO terms were classified into biological process, cellular component, and molecular function (Figure 4).

### 2.7. Highly Regulated GO Terms in Biological Processes

Biological processes contained, overall, 16 DEGs, which were highly regulated in proteolysis (*p* = 6.411 × 10^−5^), 9 DEGs in the lipid metabolic process (*p* = 5.341 × 10^−3^), and 6 DEGs in the carboxylic acid metabolic process (*p* = 0.032). Hydrolase activity was the most strongly upregulated GO term, with 5 DEGs regulated, followed by 3 significantly upregulated DEGs in proteolysis (13 DEGs), lipid metabolic process (7 DEGs), and 3 DEGs in lipid biosynthetic process and aminoglycan metabolic process (*p* = 0.016 and 0.045). In comparison, examination of the downregulated GOs in biological processes has shown that a total of 4 DEGs were significantly downregulated in carboxylic acid metabolism, oxoacid metabolic process (*p* = 0.033), and organic acid metabolic method (*p* = 0.034), and 3 DEGs in proteolysis (Figure 4 and Table S9).

### 2.8. Highly Regulated GO Term in the Cellular Component

After the exposure of indoxacarb, the membrane included 22 DEGs, while the integral component, the intrinsic component, and the membrane part contained a total of 16 significant regulated DEGs (*p* = 0.021, 0.004, 0.005, and 0.016, respectively). The membrane with 16 DEGs and the integral membrane component, the intrinsic membrane component, and the membrane part with 12 DEGs showed significant upregulation. While there were 6 DEGs in the membrane, and 4 DEGs in the intrinsic and integral portion of the membrane, the membrane components were significantly downregulated (Figure 4 and Table S9).

### 2.9. Highly Regulated GO Terms in Molecular Function

In the analysis of the GO terms associated with molecular functions, 50 DEGs in catalytic activity, 24 DEGs in hydrolase activity, and 18 DEGs in catalytic activity were found to be highly regulated (*p* = 6.914 × 10^−3^, 0.0113, and 0.011, respectively). Among them, a total of 30 DEGs of catalytic activity, 18 DEGs of hydrolase activity, and 13 DEGs of catalytic activity, acting on a protein were considerably upregulated. In the meantime, 20 DEGs in catalytic activity, 6 DEGs in hydrolase activity, and 5 DEGs catalytic activity were significantly downregulated (Figure 4 and Table S9).

### 2.10. Detoxification Mechanisms Related to Genes

**Figure 5:**
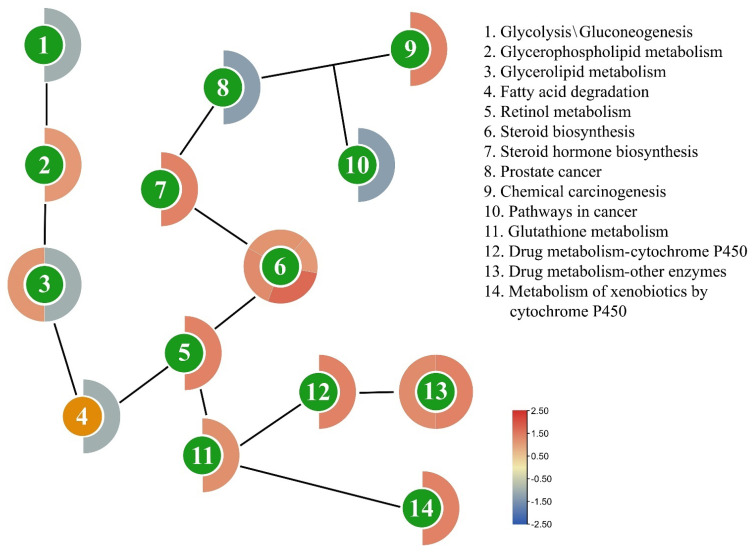
Scheme of the different pathways in the chain affected by the exposure of indoxacarb (after 24 h). The circle’s color indicates the pathway’s function, whereas the strip’s color outside the circle indicates how DEGs are regulated on a scale.

**Figure 6:**
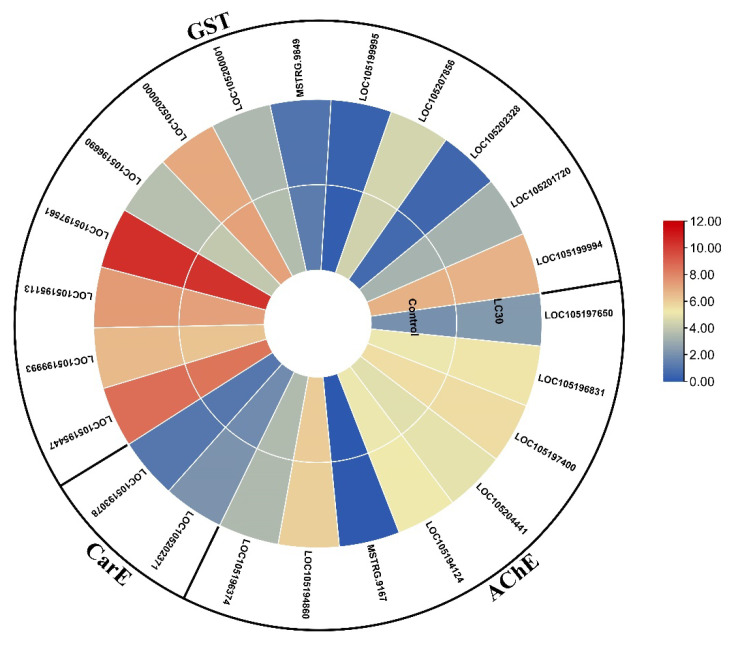
Heatmap of expression of genes related to acetylcholine esterase (AChE), carboxylesterase (CarE), and glutathione S-transferases (GSTs), based on mean FPKM values exposed to indoxacarb (after 24 h).

After 24 h of indoxacarb exposure, several metabolic pathways that are directly associated with pesticide detoxification were shown to be altered, including steroid biosynthesis (4 DEGs), other drug-metabolizing enzymes (2 DEGs), glycerolipid metabolism (2 DEGs), chemical carcinogenesis (1 DEG), drug-metabolizing cytochrome P450 (1 DEG), fatty acid degradation (1 DEG), glutathione metabolism (1 DEG), glycerophospholipid metabolism (1 DEG), glycolysis/gluconeogenesis (1 DEG), metabolism of xenobiotics by cytochrome P450 (1 DEG), pathways in cancer (1 DEG), prostate cancer (1 DEG), retinol metabolism (1 DEG), and steroid hormone biosynthesis (1 DEG). The mechanism of these pathways is depicted in Figure 5, a heatmap shows how an effect on one pathway triggers a chain reaction of genetic makeup changes. The gene expression of AChE, CarE, and GST, genes are also shown in Figure 6, and the list of genes is also provided in Table 2.

### 2.11. Validation of Transcriptome via RT-qPCR

**Figure 7:**
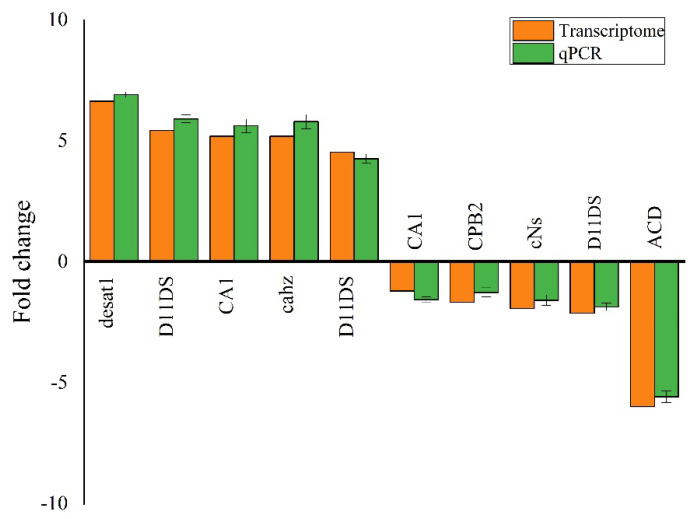
Quantitative PCR (qPCR) authentication of the DEGs associated with detoxification indoxacarb in *Solenopsis invicta*.

Ten genes were selected from various pathways to evaluate DEGs regulation and validate transcriptome data. The RT-qPCR results establish the validity of the transcriptome analysis (Figure 7; Tables S1 and S2).

## 3. Discussion

The development of pesticide resistance in insect pests is a primary global concern, as various species have shown resistance to various insecticide classes [47]. Among the insecticides, indoxacarb has been widely used to suppress fire ants in China [48]. Indoxacarb is well known for its ability to manage a wide range of crop pests at low dosages [49], with underlying side effects posed on non-target organisms [50]. Indoxacarb mode of action involves the blockage of the sodium channel in the nerve cells of the target organisms, causing the midgut to break down [51]. However, due to the intensive use of synthetic insecticides, low susceptibility to insecticides has been reported in *S. invicta* [25]. In the current study, the fire ant population collected from GZ showed a 2-fold resistance to indoxacarb insecticide. Nevertheless, due to the discriminate use of indoxacarb, several studies have revealed that various insects have developed a high level of resistance to indoxacarb in recent years, such as *Plutella xylostella*, *Spodoptera frugiperda*, *Spodoptera litura*, *Spodoptera exigua*, and *Musca domestica* [19,52,53,54,55,56].

In the present study, detoxification enzyme activities, such as AChE, CarE, and GST, were determined to explore the responses of RIFA to sublethal concentrations of indoxacarb. The current study’s findings revealed that the activity of detoxification enzymes, such as AChE, CarE, and GST, increased with a corresponding increase in concentration, and the activity was significantly correlated with the sublethal dosage indoxacarb. Our results are consistent with the findings of previous studies, where the higher detoxifying activity of AChE, CarE, and GST enzymes was reported when *S. invicta*, *Helicoverpa armigera*, and *Neoseiulus californicus* were exposed to indoxacarb and fipronil insecticides [57,58,59]. The increased activity of detoxifying enzymes, such as AChE, CarE, and GST, may provide significant evidence in developing indoxacarb resistance in *Helicoverpa assulta* [60]. Gao et al. [61] discovered that CarE and GST were two of the most critical factors contributing to the indoxacarb resistance in the populations of *S. exigua*. The activity of these enzymes increases in response to indoxacarb. Similarly, Nehare et al. [62] revealed that GST participated in *P. xylostella* resistance to indoxacarb. However, the mechanism by which *S. invicta* resists indoxacarb remains unknown at the molecular level.

A comprehensive investigation of detoxification gene families included in pesticide resistance is essential in understanding the biochemical and development of insecticide resistance mechanisms in invasive insect pests [63]. This study evaluated transcriptome analysis and characterization of the gene expression related to insecticide resistance of *S. invicta* under indoxacarb stress. Based on transcriptional data, the differential expression of unigenes indicates that enriched GOs and numerous biological pathways are involved in developing pesticide resistance mechanisms, according to the findings. We examined the entire body transcriptomic response of *S. invicta* against indoxacarb to find the differentially expressed detoxifying genes and associated metabolic pathways. Between the *S. invicta* control and treated groups, a total of 158 DEGs were detected (Table S7). Among them, 100 (63.29%) DEGs were significantly upregulated, and 58 (36.70%) DEGs were significantly downregulated in the indoxacarb-treated samples compared with the control samples. The majority of the elevated genes belonged to the AChE, CarE, and GSTs families. These results correspond with those reported for *S. invicta*, *S. exigua*, and *S. frugiperda*, when exposed to fipronil, lambda-cyhalothrin, and indoxacarb [25,62,63]. In this study, a total of 23 genes from AChE, CarE, and GSTs families were commonly regulated with Log2FC values of 1.5–2 (Figure 6) in treated *S. invicta*. According to previous findings in other insects, several AChE, CarE, and GST genes were overexpression was discovered after exposure to a variety of synthetic insecticides, including indoxacarb. [61,64,65]. Therefore, our results showed that upregulation of multiples AChE, CarE, and GSTs genes are associated with indoxacarb exposure in *S. invicta*; whether these genes can actively metabolize indoxacarb insecticide requires further investigation.

Tolerance to insecticides requires not only detoxification but also decreased sensitivity and permeability at the target location. In the current study, indoxacarb treatment of *S. invicta* resulted in a decrease in the transcription of all cuticle protein genes. Previous research has shown that cuticle proteins are the first line of defense used by almost all insects to prevent hazardous chemical penetration and regulate water loss [66,67]. In contrast to our findings, several previous studies have documented the differential transcriptome level of many cuticular genes in the diversity of insects after exposure to insecticides [65,66,67,68]. The current data imply that downregulation of cuticular genes may contribute to decreased susceptibility to indoxacarb insecticide in *S. invicta*, leading to higher tolerance to insecticide-induced stress. In addition, we identified several ABC transporter genes which were mostly downregulated in indoxacarb insecticide-treated *S. invicta*. In many previous studies, it has been reported the involvement of ABC transporter genes in the transference of numerous molecules, including several xenobiotics compounds, and revealed amplified transcription levels against various groups of insecticides stress in the diversity of insects [69,70,71]. In addition, after exposure to indoxacarb insecticide, three nuclear hormone receptor genes (nr2c2ap, Ncoa5, and NCOR1) also revealed downregulated transcription levels in *S. invicta*. It has been documented that transcriptional activators are used to initiate the expression of particular metabolic enzymes with the help of these nuclear hormone receptors, which promote detoxification [72]. Our present study indicated that several annotated DEGs were animatedly intricate in major KEGG detoxification pathways, including lipoic acid metabolism, metabolic pathways, cholesterol metabolism, and fatty acid metabolism by other enzymes (Figure 5). This reveals their relationship with reducing the antagonistic effects of pesticide exposure on *S. invicta*. Due to their lipophilicity, many toxicant metabolites are challenging to eliminate from the body. They accumulate and are often incompatible with the biological processes of organisms as a result of lipophilic compounds [72,73]. Our transcriptomic data revealed that indoxacarb insecticide tolerance by *S. invicta* is not due to the regulation of a single gene but, moderately, it is a result of combined transcription regulation by multiple detoxifying genes.

## 4. Materials and Methods

### 4.1. Test Insects

The fire ant samples were collected from the insecticide application areas of Guangzhou (GZ), Heyuan (HY), Huizhou (HZ), Shenzhen (SZ), Zhongshan (ZS), Zhuhai (ZH), Dongguan (DG), and Jiangmen (JM) of Guangdong province, China (Figure S1). Twenty-seven colonies were collected from these cities. From GZ, six colonies were collected, while three were collected from other cities. Colonies were isolated and placed in plastic boxes using the water floating method [74] and kept in the laboratory at ambient temperature and relative humidity, 26 ± 1 °C, and 60 ± 3%, respectively [75]. The upper portion of the inner wall of the rearing box was covered with a mixture of talc powder and ethanol to prevent ants from escaping [76]. We provided the mealworm larvae (*Tenebrio molitor*) and water tubes containing 10% *w*/*w* sugar as a food source. Before experimentation, the colonies were kept at ambient laboratory conditions to allow the ants to become acclimated to the laboratory environment.

### 4.2. Toxicity Bioassay

The residual bioassay has been used to assess the fire ant worker’s susceptibility to indoxacarb concentrations (LC_10_, LC_30_, LC_50_, and LC_90_) [77]. A total of 3 colonies were tested, and 3 trials—each involving 30 medium-sized (4–6 mm) worker samples—were conducted for each colony in a flask under standard conditions [75]. The indoxacarb, 90% (Shandong Jingbo agrochemical Technology Co., Ltd., Binzhou, China), was used. The stock solutions (1000 μg/mL) of indoxacarb were generated in acetone, and then increased the concentrations in the geometric ratio (including control) were generated in acetone. In total, 10 indoxacarb concentrations (0.0075%, 0.01%, 0.0125%, 0.015%, 0.0175%, 0.02%, 0.0225%, 0.0250%, 0.0275%, 0.0300%) were used in bioassays. For each indoxacarb concentration, 30 workers were taken for each replication, and 3 replications were set up. Sugar water at 10% was provided in a 1.5 mL vial in each flask. The 50 mL flask was washed 3 times with water and detergent and then washed 3 times with acetone before the experiment. Polytetrafluoroethylene (PTFE) was used to paint the upper part of 50 mL flasks to avoid ant escape. A known amount of technical grade active ingredients of indoxacarb was completely dissolved in acetone and the nine serial dilutions were prepared. A glass pipette was used to transfer 1 mL of each pesticide–acetone solution to a glass flask. After adding the solution, the vials were rolled slowly until dry. The insecticide residue was evenly dispersed on the inside surface after evaporating from the acetone solvent. Workers were transferred into the flask using a glass stirring rod to avoid injury. Then, 30 ant workers were placed in the treated flask—10 treatments were used, along with 1 control (acetone (99.5%) Guangzhou Guangshi reagent Technology Co., Ltd., Guangzhou, China). The ants were examined, and mortality was recorded after 24 h. An ant was assumed dead if it did not move when poked with a needle. Each trial included a control in which ants were placed in a flask containing only acetone. The flasks were kept in the rearing laboratory at a temperature of 25 ± 1 °C and relative humidity of 60 ± 3%. After 24 h of insecticide treatment, surviving *S. invicta* workers were shifted into centrifuge tubes, instantly frozen by liquid nitrogen, and stored at −80 °C for other experiments.

### 4.3. Enzyme Assay

The samples (10–12 workers) treated with LC_30_ and control were weighed before homogenization. The fire ant workers were uniformed at room temperature with ice-cold 0.05 M sodium phosphate buffer (pH 7.3). At 4 °C, homogenized RIFA samples were centrifugated at 12,000 rpm for 10 min. The supernatant was extracted, transferred into new tubes, and centrifuged at 12,000 rpm for 15 min. The last supernatants were used for various enzyme analyses. The activity of the detox enzyme (AChE, CarE, GST) was identified by commercially available kits bought from the Bioengineering Research Institute of Nanjing Jiancheng, Nanjing City, China. The manufacturer’s instructions were followed. Bio-Rad spectrophotometer (iMark) (OSAKA, Japan) used at absorbance light of 412 and 450 nm wavelength (according to the manufacturer’s protocol). Additionally, the protein concentration in the sample was measured using a Bradford assay kit acquired from Beyotime, Shanghai, China, and standardized with BSA (bovine serum albumin) [22].

### 4.4. RNA Extraction, Library Construction, and Sequencing

Following the manufacturer’s procedure, total RNA extraction from the 10–12 workers was performed via Trizol reagent kit (Invitrogen, Waltham, MA, USA). The most resistant population GZ-2 (control and LC_30_) was selected for transcriptome analysis. The quality of extracted RNA was assessed via Agilent 2100 Bioanalyzer (Agilent Technologies, Santa Clara, CA, USA) and confirmed with the electrophoresis of RNase-free agarose gel. After obtaining the complete RNA, eucaryotic mRNA was enhanced with Oligo(dT) beads, whereas prokaryotic mRNA was enhanced by Ribo-ZeroTM RNA Magnetic Kit (Epicentre, Madison, WI, USA). The enhanced mRNA was then disintegrated with fragmentation buffer and reversed translated into cDNA with primers. The dNTPs, RNase H, and DNA polymerase I were used as the starting materials for the production of second-strand cDNA. Purification was performed via Qia Quick PCR extraction kit (provided by Qiagen, Shanghai, China. Finally, the Illumina sequencing adapters were ligated. An agarose gel electrophoresis was used to identify the binding sites, then amplified using PCR and sequenced using an Illumina HiSeq2500 by Gene Denovo Biotechnology Co. (Guangzhou, China). The raw data was uploaded into the NCBI SRA database ((https://www.ncbi.nlm.nih.gov/bioproject/PRJNA797699) accessed on 21 January 2022).

### 4.5. Bioinformatics Analysis

The raw reads generated by the sequencing machines may contain low-quality bases or adapters, impairing consequent assembly and analysis. The readings were filtered further by quickp (v 0.18.0) (Chen et al., 2018) to acquire high-quality clean readings with parameters, such as deleting adapter-containing reads and eliminating the readings with above 10% unknown nucleotides (N) and over 50% low-quality (*q*-value ≤ 20) bases.

#### 4.5.1. Alignment of Ribosome RNA (rRNA)

Mapping short reads to the database of ribosomal RNA (rRNA) was accomplished using the Bowtie2 [78] alignment tool (v 2.2.8) for short reads. The rRNA-mapped readings were then eliminated. Amplification of the remaining clean reads was used to assemble and calculate gene richness. An index has been created for the reference genome, and pair-end clean reads have been mapped into the referential genome using HISAT2. 2.4 [79] with a default “rna-strandness RF” and other settings.

#### 4.5.2. Quantification of Gene Abundance

The mapped readings for every sample were gathered in a reference-based manner using StringTie (version 1.3.1) [80,81]. StringTie software was used to generate the fragment per kilobase of transcript per million mapped reads (FPKM) value for every transcriptional area to measure its variation and abundance. The following is the FPKM formula:

$$\mathrm{FPKM}=\frac{10^{6}C}{\mathrm{NL}/10^{3}}$$

PKM (A) represents gene expression, C represents the number of fragments mapped to gene A, N represents the total number of fragments mapped to reference genes, and L represents the base number on gene A.

#### 4.5.3. Differentially Expressed Genes (DEGs)

DESeq2 software (version 1.4.0) [82] was used to study RNAs differential expression between two separate groups (and by edgeR (version 3.6.0) [83] among two samples). The genes/transcripts had a false discovery rate (FDR) less than 0.05 and an absolute fold change. The gene sequences were annotated through BLASTX (version 2.2.26, NCBI, Bethesda, MA, USA).

#### 4.5.4. Relationship Analysis of Samples

##### GO Enrichment Analysis

Gene ontology (GO) [84] is an internationally recognized gene classification system that employs a dynamically updated regulated vocabulary and a well-defined concept to comprehensively describe the properties of genes and their products in any organism. The Gene ontology (GO) was divided into biological process, cellular component, and molecular function. GO’s fundamental unit is the GO term. Each GO term is associated with a specific type of ontology.

The GO enrichment analysis identifies all GO terms significantly enriched by DEGs compared with the genomic baseline and filters out DEGs that were not associated with biological activities. The gene numbers for each term were computed for each DEG. Hypergeometric tests were applied to detect significantly enriched GO terms in DEGs compared with the genome background. The following formula was used to compute *p*-value:

$$P=1-\overset{m-1}{\underset{i=0}{\sum }}\frac{\left(\begin{array}{c} M\\ i \end{array}\right)\left(\begin{array}{c} N-M\\ n-i \end{array}\right)}{\left(\begin{array}{c} N\\ n \end{array}\right)}$$

where *N* is the total number of gene annotations (including DEGs), *M* is the total number of gene annotations (including DEGs), and *M* is the total number of DEGs. The estimated *p*-value was used for FDR correction, with a threshold of FDR 0.05 being used as a cutoff. GO terms that met these criteria were designated as DEG-enriched GO terms.

##### Pathway Enrichment Analysis

Genes typically interact to perform roles in particular biological functions. Pathway analysis enables a better understanding of the biological functions of genes. The most extensive public pathway-related database is KEGG [83,85]. When comparing DEGs to the entire genome background, pathway enrichment analysis revealed considerably enhanced metabolic or signal transduction pathways. The formula is similar to the one used in GO analysis.

### 4.6. RT-qPCR for Transcriptome Validation

The transcriptome data was confirmed by choosing 5 upregulated and 5 downregulated genes. The primer pairs used are presented in (Table S1). Reverse transcription of RNA samples using EasyScript and SuperMix (Transgen Biotech, Beijing, China) was engaged for cDNA synthesis. While RT-qPCR was employed for SYBR green detection (Takara, Dalian, China) using Agilent Mx3005P as specified by the manufacturer, where rpl13 was used as the reference gene. The relative gene expression was computed using the 2-Ct technique [86].

### 4.7. Statistical Analysis

The data were analyzed by using SPSS (v22.0). The adjusted mortality was calculated using the toxicity bioassay Abbott formula [87]. The probit analysis calculated the LC_10_, LC_30_, and LC_50_ values with a 95% confidence interval (CIs). When the 95% of confidence intervals did not overlap, the LC_50_ values were significantly different. The resistance ratio is calculated using the formula below:

$$P=1-\overset{m-1}{\underset{i=0}{\sum }}\frac{\left(\begin{array}{c} M\\ i \end{array}\right)\left(\begin{array}{c} N-M\\ n-i \end{array}\right)}{\left(\begin{array}{c} N\\ n \end{array}\right)}$$

A one-way ANOVA, followed by Tukey’s test, evaluated the mean difference in enzyme activity analysis with three replications. The R studio was used to calculate the sublethal concentration-enzyme activity relation. The graphical work was carried out with Sigma plot 12.0.

## 5. Conclusions

The current study examines the risk of *S. invicta* developing resistance to indoxacarb. The resistance against indoxacarb is higher in fire ants found in Guangzhou than in other cities of Guangdong province. AChE, CarE, and GST were actively involved in indoxacarb resistance. We predicted a link between enhanced enzyme activity and resistance to indoxacarb in *S. invicta*. To better manage the development of resistance, it is necessary to utilize specific measures, such as chemical rotation. In an indoxacarb-treated population of *S. invicta*, a total of 158 DEGs were found (100 upregulated and 58 downregulated). The upregulation of numerous detoxification-related genes, GO terms, and KEGG pathways in *S. invicta* may be significant for insecticide detoxification. However, more genetic and molecular investigations will be required to confirm the current findings.

## Figures and Tables

**Table 1:** LC_10_, LC_30,_ and LC_50_ values of *Solenopsis invicta* obtained from exposure of indoxacarb.

Population	n	LC_10_ (%) (95% CL)	LC_30_ (%) (95% CL)	LC_50_ (%) (95% CL)	Slope ± SE	X^2^ (df)
GZ-1	1350	0.012 (0.010–0.013) ^a^	0.015 (0.014–0.016) ^a^	0.018 (0.016–0.019) ^a^	12.174 ± 0.0.64	24.607 (8)
GZ-2	2430	0.013 (0.011–0.014) ^ab^	0.017 (0.015–0.018) ^ab^	0.020 (0.018–0.021) ^a^	12.281 ± 0.480	42.373 (8)
HY	1200	0.005 (0.004–0.006) ^ab^	0.008 (0.007–0.009) ^ab^	0.011 (0.011–0.012) ^ab^	07.255 ± 0.518	05.280 (7)
HZ	1200	0.007 (0.005–0.008) ^ab^	0.010 (0.009–0.012) ^ab^	0.014 (0.012–0.015) ^ab^	07.552 ± 0.522	13.966 (7)
SZ	960	0.004 (0.003–0.005) ^b^	0.007 (0.006–0.008) ^b^	0.011 (0.010–0.012) ^ab^	06.439 ± 0.564	06.680 (7)
ZS	2160	0.007 (0.006–0.009) ^ab^	0.010 (0.008–0.011) ^ab^	0.012 (0.011–0.013) ^ab^	11.659 ± 0.466	33.651 (7)
ZH	2430	0.004 (0.003–0.005) ^b^	0.007 (0.005–0.008) ^b^	0.009 (0.008–0.010) ^b^	08.045 ± 0.319	42.538 (8)
DG	2160	0.007 (0.006–0.008) ^ab^	0.010 (0.008–0.010) ^ab^	0.012 (0.011–0.013) ^ab^	11.501 ± 0.464	23.284 (7)
JM	2160	0.007 (0.005–0.009) ^ab^	0.010 (0.009–0.012) ^ab^	0.013 (0.012–0.015) ^ab^	09.860 ± 0.426	44.507 (7)

The abbreviations indicate the name of collection areas; ^a,b^ values are the means of 3 replicates. The same letter indicates no significant difference between populations at 5% levels. The Kruskal–Wallis test was performed between populations within every concentrated ion, column-wise.

**Table 2:** A list of insecticide detoxification genes regulated in the transcriptional data of *Solenopsis invicta*.

Insecticide	Detoxification Enzyme Family	Total Genes	Upregulated Genes	Downregulated Genes
Indoxacarb	Acetylcholinesterase	8	6	2
	Carboxylesterase	2	2	0
	Glutathione S-transferases	13	6	7

## Data Availability

Not applicable.

## Supplementary Materials

Figure S1: Collection sites of *Solenopsis invicta* (Coloured areas with red spots), Table S1: List of forward and reverse primers used in qPCR, Table S2: List of genes of interest with fold change, Table S3: List of DEGs involving insecticide-related metabolic pathways, Table S4: List of genes and gene expression in control and after LC_30_ application specifically related to insecticide detoxification, Table S5: List of the raw and clean reads of all samples, Table S6: List of base pairs before filters and after filter along with raw and clear data, Table S7: List of significant DEGs annotations related to insecticide detoxification, Table S8: List of KEGG pathways along with upregulated and downregulated genes, Table S9: List of gene ontology (GO) terms along with up and downregulated genes.

## Sample Availability

Samples of the compounds are not available from the authors.
