# Supplementary material for: Comprehensive Detoxification Mechanism Assessment of Red Imported Fire Ant (Solenopsis invicta) against Indoxacarb
Source: Molecules. 2022 Jan 27;27(3):870. doi: 10.3390/molecules27030870 (PMC8839056; doi:10.3390/molecules27030870)
Supplement: Supplementary file 1 [file molecules-27-00870-s001.zip › molecules-1550005-supplementary.pdf]

Comprehensive detoxification mechanism assessment of red imported fire ant (*Solenopsis invicta*) against indoxacarb

Junaid Ali Siddiqui [1], Yuping Zhang [2*], Yuanyuan Luo [3], Bamisope Steve Bamisile [1], Naveed Ur Rehman [4], Waqar Islam [5,6], Muhammad Qasim [7], Qiuying Jiang [1], and Yijuan Xu [1*]

[1] Department of Entomology, South China Agricultural University, Guangzhou, 510642, China. junaidali206@scau.edu.cn; junaidali206@gmail.com (J.A.S.); bamisopebamisile@yahoo.com (B.S.B.); 958863518@qq.com (Q. J.); xuyijuan@yahoo.com (Y.X.)

[2] Plant Protection Research Institute, Guangdong Academy of Agricultural Sciences, Guangdong Provincial Key Laboratory of High Technology for Plant Protection, Guangzhou 510640, China. zhangyp@gdppri.cn (Y.Z.)

[3] Institute for the Control of Agrochemicals, Ministry of Agriculture and Rural Affairs, Beijing 100125, China. 294455581@qq.com (Y.L.)

[4] State Key Laboratory for Conservation and Utilization of Subtropical Agro-Bioresources, Guangdong Provincial Key Laboratory of Plant Molecular Breeding, South China Agricultural University, Guangzhou 510642, China; naveed@scau.edu.cn (N.U.R.)

[5] State Key Laboratory of Desert and Oasis Ecology, Xinjiang Institute of Ecology and Geography, Chinese Academy of Sciences, Urumqi 830011, China; Ddoapsial@yahoo.com

[6] University of Chinese Academy of Science, Beijing 100049, China.

[7] Department of Agriculture and Forestry, Kohsar University Murree, 47150, Punjab, Pakistan. mqasim@kum.edu.cn (M.Q.)

* Correspondence: xuyijuan@yahoo.com; zhangyp@gdppri.cn

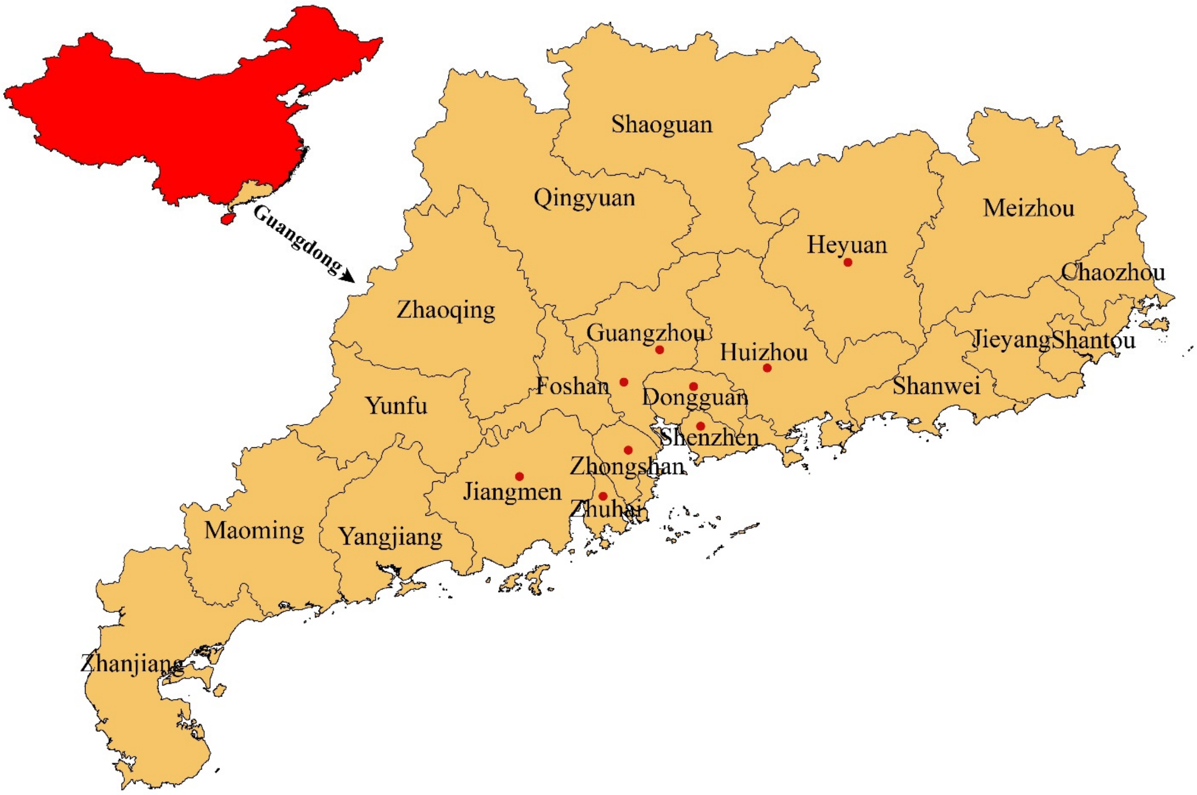

Figure S1. Collection sites of *Solenopsis invicta* (Coloured areas with red spots).

Table S1. List of forward and reverse primers used in qPCR

Genes	NR ID	Forward primer	Reverse primer
LOC105194093 (desat1)	XM_026137651	TGCAATAAAAACCGAAAAGT	TTAACCCGGAAGATACACAG
LOC105197000 (D11DS)	XM_039449512	CAAGATCGGCTGGGCCTACG	CCTTGGTGTGGTGGTGGGTC
LOC105201459 (CA1)	XM_011169477	TGGAGGGAGGAGGAGCCCTA	CGATCTGGCTGCCGTAGTGG
LOC105201461 (cahz)	XM_011169479	CGAGTGCCTGAAGCAGAGGG	GGGATCTTGGTCTCGCTGCC
LOC105200011 (D11DS)	XM_026132620	TGTACGTGATCGGCGGCTTC	TCCTGTGGTCCCTCACCCAG
LOC105201491 (CA1)	XM_011169518	ACGTGGTGAGCAACCTGTGG	CTCCTGCTGCACGATCCAGG
LOC105194705 (CPB2)	XM_011159765	TGGTGTTCGTGGTGGACGTG	AAGATGTCGGCGATGCTGGG
LOC105194841 (cNs)	XM_039449217	CGACCTGGTGATCATGCCCC	GCTCGATGGGGTTCAGGTCG
LOC105200010 (D11DS)	XM_039449353	CCGACGTGAAGGAGAAGGGC	GCTCGGCGGTCTTGTAGTCC
LOC105200009 (ACD)	XM_011167361	CTTCAAGGACGGCACCCTGG	GGGGGCCACTTGCTGATCTC

Table S2. List of genes of interest with fold change.

Gene of Interest	Gene ID	log2 (fold change)
desat1	LOC105194093	6.64385619
D11DS	LOC105197000	5.437405312
CA1	LOC105201459	5.196397213
cahz	LOC105201461	5.196397213
D11DS	LOC105200011	4.544320516
CA1	LOC105201491	-1.204358499
CPB2	LOC105194705	-1.670898387
cNs	LOC105194841	-1.925999419
D11DS	LOC105200010	-2.115477217
ACD	LOC105200009	-5.984893108

Table S3. List of DEGs involving insecticide-related metabolic pathways

Gene ID	Gene description	Fold change
MSTRG.9167	LOW QUALITY PROTEIN: carboxylesterase 4A-like [Ooceraea biroi]	5.644
LOC105197650	carboxylesterase 5A [Source:RefSeq mRNA;Acc:XM_011164120.1]	0.426
LOC105196831	liver carboxylesterase-like [Source:RefSeq mRNA;Acc:XM_011162963.1]	0.217
LOC105204441	venom carboxylesterase-6-like [Source:RefSeq mRNA;Acc:XM_011173516.1]	0.125
LOC105194124	venom carboxylesterase-6-like [Source:RefSeq mRNA;Acc:XM_011158865.1]	0.065
LOC105197400	venom carboxylesterase-6, transcript variant X1 [Source:RefSeq mRNA;Acc:XM_011163721.1]	0.056
LOC105196374	liver carboxylesterase 2-like [Source:RefSeq mRNA;Acc:XM_011162296.1]	-0.052
LOC105194860	venom carboxylesterase-6-like [Source:RefSeq mRNA;Acc:XM_011159974.1]	-0.089
LOC105202371	acetylcholinesterase-like, transcript variant X1 [Source:RefSeq mRNA;Acc:XM_011170846.1]	0.304
LOC105193078	acetylcholinesterase-like [Source:RefSeq mRNA;Acc:XM_011157408.1]	0.940
LOC105199995	glutathione S-transferase-like [Source:RefSeq mRNA;Acc:XM_011167343.1]	0.886
LOC105199993	glutathione S-transferase-like [Source:RefSeq mRNA;Acc:XM_011167341.1]	0.280
LOC105195447	glutathione S-transferase theta-1-like, transcript variant X5 [Source:RefSeq mRNA;Acc:XM_011160838.1]	0.212
LOC105195113	microsomal glutathione S-transferase 1-like [Source:RefSeq mRNA;Acc:XM_011160360.1]	0.148
LOC105197561	glutathione S-transferase-like [Source:RefSeq mRNA;Acc:XM_011163987.1]	0.087
LOC105207856	glutathione S-transferase D4-like [Source:RefSeq mRNA;Acc:XM_011177540.1]	0.085
LOC105201720	glutathione S-transferase 1-like [Source:RefSeq mRNA;Acc:XM_011169880.1]	-0.017
LOC105199994	glutathione S-transferase-like [Source:RefSeq mRNA;Acc:XM_011167342.1]	-0.051
LOC105200001	glutathione S-transferase-like [Source:RefSeq mRNA;Acc:XM_011167348.1]	-0.178
LOC105200000	glutathione S-transferase-like [Source:RefSeq mRNA;Acc:XM_011167347.1]	-0.188
LOC105202328	glutathione S-transferase-like [Source:RefSeq mRNA;Acc:XM_011170779.1]	-0.287
LOC105196690	glutathione S-transferase C-terminal domain-containing protein homolog [Source:RefSeq mRNA;Acc:XM_011162741.1]	-0.310
MSTRG.9849	glutathione S-transferase 1-like [*Solenopsis invicta*]	-0.486

Table S4. List of genes and gene expression in control and after LC_{30} application specifically related to insecticide detoxification.

SR. No	Gene ID	NR Discription	FPKM Control	FPKM LC30
**Acetylcholinesterase**				
1	MSTRG.9167	LOW QUALITY PROTEIN: carboxylesterase 4A-like [Ooceraea biroi]	0.001	0.05
2	LOC105197650	carboxylesterase 5A [Source:RefSeq mRNA;Acc:XM_011164120.1]	3.01	4.043333
3	LOC105196831	liver carboxylesterase-like [Source:RefSeq mRNA;Acc:XM_011162963.1]	34.11	39.64667
4	LOC105204441	venom carboxylesterase-6-like [Source:RefSeq mRNA;Acc:XM_011173516.1]	27.69667	30.19333
5	LOC105194124	venom carboxylesterase-6-like [Source:RefSeq mRNA;Acc:XM_011158865.1]	34.07	35.64
6	LOC105197400	venom carboxylesterase-6, transcript variant X1 [Source:RefSeq mRNA;Acc:XM_011163721.1]	45.75	47.57667
7	LOC105196374	liver carboxylesterase 2-like [Source:RefSeq mRNA;Acc:XM_011162296.1]	10.63	10.25333
8	LOC105194860	venom carboxylesterase-6-like [Source:RefSeq mRNA;Acc:XM_011159974.1]	65.45	61.54333
**Carboxylesterase**				
1	LOC105202371	acetylcholinesterase-like, transcript variant X1 [Source:RefSeq mRNA;Acc:XM_011170846.1]	2.55	3.423333
2	LOC105193078	acetylcholinesterase-like [Source:RefSeq mRNA;Acc:XM_011157408.1]	1.2	1.21
Glutathione-S-transferase				
1	LOC105199995	glutathione S-transferase-like [Source:RefSeq mRNA;Acc:XM_011167343.1]	0.153333	0.283333
2	LOC105199993	glutathione S-transferase-like [Source:RefSeq mRNA;Acc:XM_011167341.1]	77.78	94.47
3	LOC105195447	glutathione S-transferase theta-1-like, transcript variant X5 [Source:RefSeq mRNA;Acc:XM_011160838.1]	355.5933	411.7667
4	LOC105195113	microsomal glutathione S-transferase 1-like [Source:RefSeq mRNA;Acc:XM_011160360.1]	152.4767	168.99
5	LOC105197561	glutathione S-transferase-like [Source:RefSeq mRNA;Acc:XM_011163987.1]	1306.023	1387.273
6	LOC105207856	glutathione S-transferase D4-like [Source:RefSeq mRNA;Acc:XM_011177540.1]	19.97667	21.18333
7	LOC105201720	glutathione S-transferase 1-like [Source:RefSeq mRNA;Acc:XM_011169880.1]	8.626667	8.526667
8	LOC105199994	glutathione S-transferase-like [Source:RefSeq mRNA;Acc:XM_011167342.1]	111.0567	107.2067
9	LOC105200001	glutathione S-transferase-like [Source:RefSeq mRNA;Acc:XM_011167348.1]	11.31333	10.00333
10	LOC105200000	glutathione S-transferase-like [Source:RefSeq mRNA;Acc:XM_011167347.1]	150.24	131.8467
11	LOC105202328	glutathione S-transferase-like [Source:RefSeq mRNA;Acc:XM_011170779.1]	0.573333	0.47
12	LOC105196690	glutathione S-transferase C-terminal domain-containing protein homolog [Source:RefSeq mRNA;Acc:XM_011162741.1]	15.16	12.22667
13	MSTRG.9849	glutathione S-transferase 1-like [Solenopsis invicta]	1.456667	1.04

Table S5. List of the raw and clean reads of all samples.

Sample	Raw Read	Clean Read
Ind-C1	48447166	48347126
Ind-C2	46554290	46447634
Ind-C3	47653952	47528044
**Average**	**47551802.67**	**47440934.67**
Ind-30-1	43807734	43711698
Ind-30-2	51239578	51134350
Ind-30-3	58207038	57965708
**Average**	**51084783.33**	**50937252**

Table S6. List of base pairs before filters and after filter along with raw and clear data

**Sample**	**RawData(bp)**	**BF_Q30(%)**	**BF_GC(%)**	**CleanData(bp)**	**AF_Q30 (%)**	**AF_GC (%)**
**Ind-C1**	7267074900	6854514003 (94.32%)	2721495543 (37.45%)	7225022371	6823226712 (94.44%)	2699768226 (37.37%)
**Ind-C2**	6983143500	6563440512 (93.99%)	2681338745 (38.40%)	6931556820	6524564819 (94.13%)	2654540978 (38.30%)
**Ind-C3**	7148092800	6747257693 (94.39%)	3196895986 (44.72%)	7098409469	6710169406 (94.53%)	3169643175 (44.65%)
**Ind-L30-1**	6571160100	6209764858 (94.50%)	2652270388 (40.36%)	6527487090	6176793042 (94.63%)	2629368120 (40.28%)
**Ind-L30-2**	7685936700	7262561510 (94.49%)	3111302556 (40.48%)	7626863460	7216886462 (94.62%)	3080613856 (40.39%)
**Ind-L30-3**	8731055700	8142416305 (93.26%)	3490602494 (39.98%)	8646724452	8082609363 (93.48%)	3447643516 (39.87%)

Table S7. List of significant DEGs annotations related to insecticide detoxification.

ID	Description	log2(fc)	P-Value
LOC105198422	XP_011163427.1 uncharacterized protein LOC105198422 [*Solenopsis invicta*]	-2.00012322	1.28E-47
LOC105198854	mpv17-like protein 2 [Source:RefSeq mRNA;Acc:XM_011165698.1]	1.51798535<br>2	1.59E-39
LOC105194589	synaptic vesicle glycoprotein 2B-like [Source:RefSeq mRNA;Acc:XM_011159572.1]	2.43808553<br>8	7.52E-32
LOC105201375	myc-associated zinc finger protein [Source:RefSeq mRNA;Acc:XM_011169370.1]	-2.0349442	4.95E-27
LOC105204027	heat shock protein 83 [Source:RefSeq mRNA;Acc:XM_011173022.1]	-1.33403097	8.44E-27
LOC105195283	alkaline phosphatase 4-like, transcript variant X2 [Source:RefSeq mRNA;Acc:XM_011160655.1]	-3.60019227	8.86E-19
LOC105197404	cytochrome P450 6k1-like, transcript variant X3 [Source:RefSeq mRNA;Acc:XM_011163732.1]	1.59493745<br>3	1.60E-12
LOC105203274	growth/differentiation factor 8, transcript variant X2 [Source:RefSeq mRNA;Acc:XM_011172052.1]	1.24125408<br>6	3.42E-12
LOC105194316	XP_011157475.1 uncharacterized protein LOC105194316 isoform X1 [*Solenopsis invicta*]	-1.04578417	8.90E-12
LOC105200780	UDP-glucuronosyltransferase 3A1-like [Source:RefSeq mRNA;Acc:XM_011168499.1]	1.63231216<br>1	3.41E-11
LOC105200109	rhythmically expressed gene 5 protein [Source:RefSeq mRNA;Acc:XM_011167503.1]	1.13564856<br>4	7.19E-11
LOC105198588	lipase 3-like [Source:RefSeq mRNA;Acc:XM_011165343.1]	1.18275490<br>2	1.50E-10
LOC105193464	XP_011156219.1 uncharacterized protein LOC105193464 [*Solenopsis invicta*]	2.14457557<br>2	1.55E-10
LOC105207901	XP_011175889.1 uncharacterized protein LOC105207901 [*Solenopsis invicta*]	1.65743561<br>2	2.19E-10
LOC105194676	voltage-dependent L-type calcium channel subunit beta-2, transcript variant X5 [Source:RefSeq mRNA;Acc:XM_011159729.1]	1.17618878<br>7	9.90E-10
LOC105196774	XP_011161179.1 uncharacterized protein LOC105196774 [*Solenopsis invicta*]	-1.31403627	2.38E-09
LOC105200857	XP_011166938.1 uncharacterized protein LOC105200857 [*Solenopsis invicta*]	-1.49540839	3.16E-09
LOC105194877	serine/threonine-protein kinase grp, transcript variant X2 [Source:RefSeq mRNA;Acc:XM_011160001.1]	-1.11773485	3.26E-09

LOC105202983	XP_011169982.1 uncharacterized protein LOC105202983, partial [*Solenopsis invicta*]	-1.92625306	3.88E-09
LOC105204662	XP_011172130.1 uncharacterized protein LOC105204662 [*Solenopsis invicta*]	1.30976545<br>6	4.04E-09
LOC105207862	nuclear pore complex protein Nup160 homolog [Source:RefSeq mRNA;Acc:XM_011177508.1]	-1.12418917	4.26E-09
LOC105199272	RNA-directed DNA polymerase from mobile element jockey-like, transcript variant X2 [Source:RefSeq mRNA;Acc:XM_011166279.1]	1.59941816<br>4	4.55E-09
LOC105203338	XP_025994720.1 uncharacterized protein LOC105203338 [*Solenopsis invicta*]	1.29142058<br>9	5.13E-09
LOC105200010	acyl-CoA Delta(11) desaturase, transcript variant X1 [Source:RefSeq mRNA;Acc:XM_011167362.1]	-1.67089839	5.35E-09
LOC105196458	DNA ligase 1-like [Source:RefSeq mRNA;Acc:XM_011162405.1]	1.34203314<br>8	7.46E-09
LOC105201911	histone-lysine N-methyltransferase SETMAR-like [Source:RefSeq mRNA;Acc:XM_011170190.1]	1.47164986<br>7	1.04E-08
LOC105197030	XP_025995677.1 uncharacterized protein LOC105197030 isoform X2 [*Solenopsis invicta*]	-1.74723393	8.54E-08
LOC105202384	cysteine sulfinic acid decarboxylase [Source:RefSeq mRNA;Acc:XM_011170867.1]	-1.17191398	1.36E-07
LOC105205726	dorsalin-1-like [Source:RefSeq mRNA;Acc:XM_011175216.1]	1.57571018<br>7	1.49E-07
LOC105192918	fatty acid synthase-like [Source:RefSeq mRNA;Acc:XM_011157205.1]	-1.3014719	1.58E-07
LOC105201875	XP_011168444.1 uncharacterized protein LOC105201875 [*Solenopsis invicta*]	1.76953057<br>4	2.74E-07
NC_014672.1-<br>ND3	ND3 NADH dehydrogenase subunit 3 [*Solenopsis invicta* (red fire ant) -	-1.09665645	2.80E-07
LOC105193936	aminopeptidase N-like [Source:RefSeq mRNA;Acc:XM_011158626.1]	1.46200715<br>7	5.74E-07
LOC105204328	fatty acid synthase-like [Source:RefSeq mRNA;Acc:XM_011173377.1]	-1.32297542	6.33E-07
LOC105207756	XP_011175696.1 uncharacterized protein LOC105207756 isoform X1 [*Solenopsis invicta*]	1.29005445<br>3	7.97E-07
LOC105196694	probable salivary secreted peptide [Source:RefSeq mRNA;Acc:XM_011162746.1]	1.56452459	8.41E-07
LOC105200309	peritrophin-1-like [Source:RefSeq mRNA;Acc:XM_011167786.1]	1.72938107<br>4	1.05E-06
LOC105206355	XP_025994997.1 uncharacterized protein LOC105206355 [*Solenopsis invicta*]	1.97949232<br>2	1.13E-06
LOC105198220	esterase E4-like [Source:RefSeq mRNA;Acc:XM_011164877.1]	1.41799081<br>7	1.45E-06
LOC105195396	PBAN-type neuropeptides-like [Source:RefSeq mRNA;Acc:XM_011160774.1]	1.11091882<br>6	1.49E-06
LOC105205291	XP_025986450.1 putative aminopeptidase-2 [*Solenopsis invicta*]	1.23062628<br>5	1.95E-06

MSTRG.17342	oxidoreductase ptaE-like [*Solenopsis invicta*]	1.50502086<br>1	2.31E-06
LOC105200093	chymotrypsin-2-like [Source:RefSeq mRNA;Acc:XM_011167468.1]	2.27623176	2.44E-06
LOC105197108	aminopeptidase N [Source:RefSeq mRNA;Acc:XM_011163339.1]	1.84957202<br>4	2.48E-06
LOC105201710	cytochrome b5-like, transcript variant X4 [Source:RefSeq mRNA;Acc:XM_011169860.1]	1.41078564<br>1	2.55E-06
LOC105202141	homeobox protein Nkx-2.5-like, transcript variant X1 [Source:RefSeq mRNA;Acc:XM_011170530.1]	1.60660015	2.97E-06
LOC105202620	XP_025996942.1 endothelin-converting enzyme 1-like [*Solenopsis invicta*]	-1.83995959	3.10E-06
LOC105193305	chymotrypsin-1-like [Source:RefSeq mRNA;Acc:XM_011157712.1]	2.15183275<br>3	3.14E-06
LOC105196223	ATP-dependent RNA helicase ddx24 [Source:RefSeq mRNA;Acc:XM_011162031.1]	-1.29849453	3.25E-06
LOC105205908	chymotrypsin-2-like [Source:RefSeq mRNA;Acc:XM_011175449.1]	1.58146526<br>7	3.52E-06
LOC105196063	4-coumarate--CoA ligase 1-like, transcript variant X1 [Source:RefSeq mRNA;Acc:XM_011161786.1]	1.77495252<br>4	4.99E-06
LOC105196697	probable salivary secreted peptide [Source:RefSeq mRNA;Acc:XM_011162749.1]	1.47706675	5.27E-06
LOC105193271	chymotrypsin-1-like, transcript variant X2 [Source:RefSeq mRNA;Acc:XM_011157653.1]	1.25569843	6.57E-06
LOC105208007	15-hydroxyprostaglandin dehydrogenase [NAD(+)]-like [Source:RefSeq mRNA;Acc:XM_011177726.1]	-1.06959199	7.07E-06
LOC105204092	tetraspanin-1-like, transcript variant X1 [Source:RefSeq mRNA;Acc:XM_011173138.1]	1.69214623<br>5	8.91E-06
LOC105196696	probable salivary secreted peptide [Source:RefSeq mRNA;Acc:XM_011162748.1]	1.51678452<br>8	9.30E-06
LOC105196469	protein mesh, transcript variant X2 [Source:RefSeq mRNA;Acc:XM_011162424.1]	1.12887090<br>5	1.16E-05
LOC105201877	XP_011168446.1 uncharacterized protein LOC105201877 [*Solenopsis invicta*]	1.30335186<br>1	1.16E-05
LOC105203737	lipoyltransferase 1, mitochondrial [Source:RefSeq mRNA;Acc:XM_011172620.1]	-1.14692649	1.26E-05
LOC105207450	XP_011175203.1 uncharacterized protein LOC105207450 [*Solenopsis invicta*]	1.02958760<br>1	1.37E-05
LOC105202179	fatty acid synthase-like [Source:RefSeq mRNA;Acc:XM_011170571.1]	-1.52048041	1.49E-05
LOC105199036	odorant receptor coreceptor [Source:RefSeq mRNA;Acc:XM_011165941.1]	1.74293949<br>5	1.61E-05
LOC105194954	COUP transcription factor 2-like [Source:RefSeq mRNA;Acc:XM_011160090.1]	1.96736279<br>2	1.86E-05
LOC105199217	sulfotransferase 4A1-like, transcript variant X2 [Source:RefSeq mRNA;Acc:XM_011166200.1]	1.04004226<br>9	2.02E-05
LOC105200310	XP_025995879.1 uncharacterized protein LOC105200311 [*Solenopsis invicta*]	1.46299414<br>4	2.05E-05

LOC105197985	chymotrypsin-1 [Source:RefSeq mRNA;Acc:XM_011164597.1]	1.310706246	2.24E-05
LOC105199250	XP_025993743.1 mucin-3A [*Solenopsis invicta*]	-3.7548875	2.45E-05
LOC105200663	DDB1- and CUL4-associated factor 8-like, transcript variant X1 [Source:RefSeq mRNA;Acc:XM_011168312.1]	1.418978215	2.58E-05
LOC105205233	histone-lysine N-methyltransferase SMYD3-like, transcript variant X2 [Source:RefSeq mRNA;Acc:XR_851275.1]	1.474584229	2.73E-05
LOC105193841	alpha-glucosidase-like [Source:RefSeq mRNA;Acc:XM_011158466.1]	1.703984889	2.80E-05
LOC105192910	acyl-CoA Delta(11) desaturase-like [Source:RefSeq mRNA;Acc:XM_011157198.1]	2.051632768	2.86E-05
LOC105197056	solute carrier family 2, facilitated glucose transporter member 8 [Source:RefSeq mRNA;Acc:XM_011163268.1]	1.066250221	2.90E-05
LOC105196621	zinc carboxypeptidase-like [Source:RefSeq mRNA;Acc:XM_011162639.1]	-3.98334266	3.12E-05
LOC105206234	chymotrypsin-1-like [Source:RefSeq mRNA;Acc:XM_011175743.1]	1.752484206	3.54E-05
LOC105196064	luciferin 4-monooxygenase-like [Source:RefSeq mRNA;Acc:XM_011161789.1]	1.210265785	4.69E-05
LOC105199102	venom metalloproteinase 3-like [Source:RefSeq mRNA;Acc:XM_011166059.1]	-3.16474476	4.75E-05
LOC105193273	chymotrypsin-2-like [Source:RefSeq mRNA;Acc:XM_011157656.1]	1.349862911	4.94E-05
LOC105202067	XP_025988188.1 uncharacterized protein LOC105202067 isoform X1 [*Solenopsis invicta*]	1.136934276	6.06E-05
LOC105206478	aminopeptidase N-like [Source:RefSeq mRNA;Acc:XM_011175971.1]	1.759586389	6.46E-05
LOC105208221	XP_025991122.1 uncharacterized protein LOC105208221 isoform X2 [*Solenopsis invicta*]	-1.30666134	7.01E-05
LOC105200436	probable serine/threonine-protein kinase kinX [Source:RefSeq mRNA;Acc:XM_011167990.1]	1.052741462	7.16E-05
LOC105203249	XP_011170328.1 protein snakeskin [*Solenopsis invicta*]	1.087395296	7.94E-05
MSTRG.15985	PREDICTED: X-ray radiation resistance-associated protein 1-like isoform X1 [Amphimedon queenslandica]-	1.231381205	8.05E-05
LOC105196158	peptidoglycan-recognition protein 1-like, transcript variant X3 [Source:RefSeq mRNA;Acc:XM_011161931.1]	1.850665767	8.90E-05
LOC105197690	tRNA pseudouridine synthase-like 1 [Source:RefSeq mRNA;Acc:XM_011164178.1]	-1.02502079	9.44E-05
LOC105203835	lipase 3-like, transcript variant X2 [Source:RefSeq mRNA;Acc:XM_011172749.1]	1.416586158	0.000108707
LOC105206762	KYM96908.1 hypothetical protein ALC62_12418 [Cyphomyrmex costatus]	-1.58343987	0.000116541

LOC105193038	cilia- and flagella-associated protein 57 [Source:RefSeq mRNA;Acc:XM_011157359.1]	-1.19286936	0.000117887
LOC105193825	XP_011156738.1 uncharacterized protein LOC105193825 [*Solenopsis invicta*]	1.143957595	0.00012018
LOC105205116	KMQ86092.1 pol polyprotein [Lasius niger]	-1.2257802	0.000144641
LOC105205254	probable queuine tRNA-ribosyltransferase, transcript variant X1 [Source:RefSeq mRNA;Acc:XM_011174584.1]	-1.04350164	0.000148683
LOC105203861	microtubule-associated protein futsch-like [Source:RefSeq mRNA;Acc:XM_011172784.1]	-1.60572106	0.000153405
LOC105203631	glucose dehydrogenase [FAD, quinone]-like [Source:RefSeq mRNA;Acc:XM_011172483.1]	1.479307759	0.000167728
LOC105198257	sodium-coupled monocarboxylate transporter 1-like, transcript variant X1 [Source:RefSeq mRNA;Acc:XM_011164923.1]	-1.26859048	0.00016888
LOC105197474	protein lethal (2)denticleless, transcript variant X2 [Source:RefSeq mRNA;Acc:XM_011163856.1]	-1.11051245	0.000170293
LOC105201115	XP_011167307.1 uncharacterized protein LOC105201115 [*Solenopsis invicta*]	-1.03703869	0.000185371
LOC105204587	transient receptor potential channel pyrexia-like [Source:RefSeq mRNA;Acc:XM_011173715.1]	1.290289604	0.000202261
LOC105196270	cytochrome c oxidase subunit NDUFA4-like [Source:RefSeq mRNA;Acc:XM_011162094.1]	1.236983076	0.000216706
LOC105205655	XP_011173383.1 uncharacterized protein LOC105205655 isoform X1 [*Solenopsis invicta*]	-1.23104429	0.000236349
LOC105198898	trypsin-1-like [Source:RefSeq mRNA;Acc:XM_011165755.1]	1.57418253	0.0002486
LOC105193417	putative inorganic phosphate cotransporter [Source:RefSeq mRNA;Acc:XM_011157848.1]	1.009080008	0.00025935
LOC105206675	fatty acid synthase-like [Source:RefSeq mRNA;Acc:XM_011176157.1]	-1.01767189	0.000262129
LOC105200664	DDB1- and CUL4-associated factor 8-like, transcript variant X2 [Source:RefSeq mRNA;Acc:XM_011168315.1]	1.468148836	0.000265761
LOC105192853	homeobox protein Hox-B1a [Source:RefSeq mRNA;Acc:XM_011157113.1]	1.543823806	0.000292309
LOC105203371	putative aldehyde dehydrogenase family 7 member A1 homolog [Source:RefSeq mRNA;Acc:XM_011172161.1]	-1.02932764	0.000296245
LOC105193131	protein phosphatase 1 regulatory subunit 3C-B [Source:RefSeq mRNA;Acc:XM_011157475.1]	-1.67542211	0.000310687
LOC105196048	putative leucine-rich repeat-containing protein DDB_G0290503, transcript variant X1 [Source:RefSeq mRNA;Acc:XM_011161769.1]	-1.43234036	0.000353264
LOC105193432	acetyl-CoA carboxylase [Source:RefSeq mRNA;Acc:XM_011157904.1]	-1.04436335	0.000358827
LOC105199969	COMM domain-containing protein 8-like [Source:RefSeq mRNA;Acc:XM_011167306.1]	-1.12808613	0.00036772

LOC105203377	XP_011170469.1 uncharacterized protein LOC105203377 [*Solenopsis invicta*]	1.289686862	0.000369172
LOC105207509	aquaporin AQPAe.a-like, transcript variant X1 [Source:RefSeq mRNA;Acc:XM_011177017.1]	1.06542921	0.00038357
MSTRG.12133	Putative ATP-dependent RNA and DNA helicase-	1.170630994	0.000384511
LOC105202163	titin-like [Source:RefSeq mRNA;Acc:XM_011170554.1]	-1.34264304	0.000411629
LOC105203816	MFS-type transporter SLC18B1-like [Source:RefSeq mRNA;Acc:XM_011172728.1]	-1.36872231	0.000471694
MSTRG.10354	kinectin isoform X9 [*Solenopsis invicta*]	-1.19236495	0.000479259
LOC105202373	XP_011169150.1 uncharacterized protein LOC105202373 [*Solenopsis invicta*]	1.369605188	0.000491913
LOC105207917	ceramide phosphoethanolamine synthase-like [Source:RefSeq mRNA;Acc:XM_011177609.1]	1.537811407	0.000531738
LOC105196039	XP_011879492.1 PREDICTED: uncharacterized protein LOC105568426 isoform X1 [Vollenhovia emeryi]	1.344813104	0.000535431
LOC105194306	myb-like protein V [Source:RefSeq mRNA;Acc:XM_011159157.1]	-2.26282993	0.000548591
LOC105203247	XP_011170325.1 uncharacterized protein LOC105203247 isoform X1 [*Solenopsis invicta*]	1.292697384	0.000556009
LOC105201798	XP_011168300.1 uncharacterized protein LOC105201798 [*Solenopsis invicta*]	1.127647823	0.000724341
LOC105203836	lipase 3-like, transcript variant X1 [Source:RefSeq mRNA;Acc:XM_011172751.1]	2.297680549	0.000735173
LOC105203220	XP_025991335.1 uncharacterized protein LOC113003954, partial [*Solenopsis invicta*]	1.42103624	0.000737935
LOC105195412	uricase [Source:RefSeq mRNA;Acc:XM_011160791.1]	-1.02762754	0.000761684
LOC105203831	protein takeout-like [Source:RefSeq mRNA;Acc:XM_011172745.1]	-1.33029646	0.000771725
MSTRG.18297	PREDICTED: fatty acid synthase [Wasmannia auropunctata]	1.193172282	0.000795024
LOC105196519	sterol O-acyltransferase 1-like [Source:RefSeq mRNA;Acc:XM_011162492.1]	1.090522641	0.000817755
LOC105196609	glyoxylate reductase/hydroxypyruvate reductase-like [Source:RefSeq mRNA;Acc:XM_011162628.1]	1.120836776	0.0008271
LOC105201912	XP_1052011912 uncharacterized protein LOC105201912	2.551698215	0.000867783
LOC105198307	histone-lysine N-methyltransferase ASHR1-like [Source:RefSeq mRNA;Acc:XM_011164973.1]	1.056955885	0.000906834

LOC105204002	phosphatidate phosphatase LPIN2, transcript variant X4 [Source:RefSeq mRNA;Acc:XM_011172979.1]	1.074172874	0.000929729
LOC105197135	XP_025995110.1 zinc finger CCCH domain-containing protein 13 isoform X1 [*Solenopsis invicta*]	1.478520803	0.001002311
MSTRG.12889	PREDICTED _uncharacterized protein -	1.145351386	0.001125349
LOC105195766	TGZ50093.1 Mitoferrin-2 [Temnothorax longispinosus]	1.001071441	0.001133342
LOC105200774	ornithine decarboxylase 2-like [Source:RefSeq mRNA;Acc:XM_011168485.1]	1.271545906	0.001142655
LOC105197687	dual specificity protein phosphatase 19 [Source:RefSeq mRNA;Acc:XM_011164175.1]	-1.1250686	0.001171957
LOC105193036	pre-mRNA-splicing factor 38A [Source:RefSeq mRNA;Acc:XM_011157356.1]	-1.03306475	0.00119464
LOC105205814	origin recognition complex subunit 2 [Source:RefSeq mRNA;Acc:XM_011175338.1]	-1.20984717	0.00128233
LOC105193964	myoblast determination protein 1 homolog [Source:RefSeq mRNA;Acc:XM_011158658.1]	1.262738499	0.001307688
LOC105205624	XP_011173334.2 uncharacterized protein LOC105205624 [*Solenopsis invicta*]	-1.54252723	0.001309994
LOC105206141	cytosolic 10-formyltetrahydrofolate dehydrogenase [Source:RefSeq mRNA;Acc:XM_011175662.1]	-3.22082461	0.001354931
LOC105202300	ras GTPase-activating-like protein IQGAP1 [Source:RefSeq mRNA;Acc:XM_011170747.1]	-1.44835757	0.001366079
LOC105199942	hydroxylysine kinase [Source:RefSeq mRNA;Acc:XM_011167275.1]	1.133054191	0.001442621
LOC105205979	glucose dehydrogenase [FAD, quinone]-like [Source:RefSeq mRNA;Acc:XM_011175512.1]	-1.32251088	0.001536541
LOC105194706	transmembrane protease serine 9-like [Source:RefSeq mRNA;Acc:XM_011159766.1]	1.297217863	0.001552805
LOC105200330	synaptic vesicular amine transporter, transcript variant X1 [Source:RefSeq mRNA;Acc:XM_011167811.1]	1.687095286	0.001656534
LOC105198569	XP_011163623.1 uncharacterized protein LOC105198569 [*Solenopsis invicta*]	1.010929731	0.001681426
LOC105204001	XP_011171278.1 uncharacterized protein LOC105204001 [*Solenopsis invicta*]	1.32185305	0.001748608
LOC105207621	forkhead box protein D1 [Source:RefSeq mRNA;Acc:XM_011177180.1]	-1.20281688	0.001797804
LOC105201575	XP_011167928.1 uncharacterized protein LOC105201575 isoform X1 [*Solenopsis invicta*]	1.411352452	0.001870145
LOC105204314	XP_011171665.1 uncharacterized protein LOC105204314 [*Solenopsis invicta*]	1.055406299	0.001871574

LOC105200995	XP_025995748.1 ETS-related transcription factor Elf-5-like isoform X1 [*Solenopsis invicta*]	-1.07757994	0.001886399
LOC105201284	cytochrome b5-related protein-like [Source:RefSeq mRNA;Acc:XM_011169236.1]	1.957898167	0.001899187
MSTRG.941	gag-pol polyprotein precursor [Lasius niger]	-1.32332133	0.001951614
LOC105205276	alpha-catulin-like [Source:RefSeq mRNA;Acc:XM_011174620.1]	1.401362562	0.001970775
LOC105195453	protein PBDC1, transcript variant X2 [Source:RefSeq mRNA;Acc:XM_011160850.1]	-1.30014024	0.002073351
LOC105207975	regulating synaptic membrane exocytosis protein 2 [Source:RefSeq mRNA;Acc:XM_011177679.1]	1.330515591	0.002094922
LOC105195174	probable serine hydrolase [Source:RefSeq mRNA;Acc:XM_011160463.1]	1.631612594	0.002206028

Table S8. List of KEGG pathways along with up-down regulated genes

ID	num	KEGG class	ID	Description	P-value	Up	Down
Metabolic pathways	27	Metabolism	ko01100	Metabolic pathways	0.016942	15	12
Fatty acid metabolism	8	Metabolism	ko01212	Fatty acid metabolism	0.003007	2	6
Insulin signaling pathway	7	Organismal Systems	ko04910	Insulin signaling pathway	0.002419	1	6
Fatty acid biosynthesis	6	Metabolism	ko00061	Fatty acid biosynthesis	0.000978	1	5
AMPK signaling pathway	6	Environmental Information Processing	ko04152	AMPK signaling pathway	0.01799	1	5
Pyruvate metabolism	3	Metabolism	ko00620	Pyruvate metabolism	0.016328	1	2
Cell cycle	2	Cellular Processes	ko04110	Cell cycle	0.310707	0	2
Protein processing in endoplasmic reticulum	2	Genetic Information Processing	ko04141	Protein processing in endoplasmic reticulum	0.539698	0	2
Protein digestion and absorption	6	Organismal Systems	ko04974	Protein digestion and absorption	0.000755	5	1
Pancreatic secretion	6	Organismal Systems	ko04972	Pancreatic secretion	0.001574	5	1
Lysine degradation	5	Metabolism	ko00310	Lysine degradation	0.004016	4	1
Glycine, serine and threonine metabolism	2	Metabolism	ko00260	Glycine, serine and threonine metabolism	0.084269	1	1
Arginine and proline metabolism	2	Metabolism	ko00330	Arginine and proline metabolism	0.092699	1	1
Ascorbate and aldarate metabolism	2	Metabolism	ko00053	Ascorbate and aldarate metabolism	0.097003	1	1

Dilated cardiomyopathy (DCM)	2	Human Diseases	ko05414	Dilated cardiomyopathy (DCM)	0.138013	1	1
Glycerolipid metabolism	2	Metabolism	ko00561	Glycerolipid metabolism	0.142782	1	1
Hypertrophic cardiomyopathy (HCM)	2	Human Diseases	ko05410	Hypertrophic cardiomyopathy (HCM)	0.142782	1	1
Biosynthesis of unsaturated fatty acids	2	Metabolism	ko01040	Biosynthesis of unsaturated fatty acids	0.269286	1	1
Oxidative phosphorylation	2	Metabolism	ko00190	Oxidative phosphorylation	0.500546	1	1
Lipoic acid metabolism	1	Metabolism	ko00785	Lipoic acid metabolism	0.054415	0	1
Histidine metabolism	1	Metabolism	ko00340	Histidine metabolism	0.093305	0	1
Taurine and hypotaurine metabolism	1	Metabolism	ko00430	Taurine and hypotaurine metabolism	0.105915	0	1
Caffeine metabolism	1	Metabolism	ko00232	Caffeine metabolism	0.13062	0	1
Antigen processing and presentation	1	Organismal Systems	ko04612	Antigen processing and presentation	0.154653	0	1
One carbon pool by folate	1	Metabolism	ko00670	One carbon pool by folate	0.166424	0	1
Thiamine metabolism	1	Metabolism	ko00730	Thiamine metabolism	0.166424	0	1
Folate biosynthesis	1	Metabolism	ko00790	Folate biosynthesis	0.275609	0	1
p53 signaling pathway	1	Cellular Processes	ko04115	p53 signaling pathway	0.275609	0	1
IL-17 signaling pathway	1	Organismal Systems	ko04657	IL-17 signaling pathway	0.31523	0	1
Th17 cell differentiation	1	Organismal Systems	ko04659	Th17 cell differentiation	0.324797	0	1
Tryptophan metabolism	1	Metabolism	ko00380	Tryptophan metabolism	0.343539	0	1

Valine, leucine and isoleucine degradation	1	Metabolism	ko00280	Valine, leucine and isoleucine degradation	0.388184	0	1
beta-Alanine metabolism	1	Metabolism	ko00410	beta-Alanine metabolism	0.421738	0	1
Propanoate metabolism	1	Metabolism	ko00640	Propanoate metabolism	0.45348	0	1
Fatty acid degradation	1	Metabolism	ko00071	Fatty acid degradation	0.476156	0	1
Glycolysis / Gluconeogenesis	1	Metabolism	ko00010	Glycolysis / Gluconeogenesis	0.497905	0	1
Progesterone-mediated oocyte maturation	1	Organismal Systems	ko04914	Progesterone-mediated oocyte maturation	0.497905	0	1
Necroptosis	1	Cellular Processes	ko04217	Necroptosis	0.504956	0	1
NOD-like receptor signaling pathway	1	Organismal Systems	ko04621	NOD-like receptor signaling pathway	0.511909	0	1
Prostate cancer	1	Human Diseases	ko05215	Prostate cancer	0.538774	0	1
Insulin resistance	1	Human Diseases	ko04931	Insulin resistance	0.54526	0	1
Transcriptional misregulation in cancers	1	Human Diseases	ko05202	Transcriptional misregulation in cancers	0.564183	0	1
Fluid shear stress and atherosclerosis	1	Human Diseases	ko05418	Fluid shear stress and atherosclerosis	0.564183	0	1
Glucagon signaling pathway	1	Organismal Systems	ko04922	Glucagon signaling pathway	0.605377	0	1
Estrogen signaling pathway	1	Organismal Systems	ko04915	Estrogen signaling pathway	0.621837	0	1

Cellular senescence	1	Cellular Processes	ko04218	Cellular senescence	0.647781	0	1
Human immunodeficiency virus 1 infection	1	Human Diseases	ko05170	Human immunodeficiency virus 1 infection	0.698851	0	1
Viral carcinogenesis	1	Human Diseases	ko05203	Viral carcinogenesis	0.731346	0	1
Purine metabolism	1	Metabolism	ko00230	Purine metabolism	0.746275	0	1
Salmonella infection	1	Human Diseases	ko05132	Salmonella infection	0.776944	0	1
Spliceosome	1	Genetic Information Processing	ko03040	Spliceosome	0.806755	0	1
PI3K-Akt signaling pathway	1	Environmental Information Processing	ko04151	PI3K-Akt signaling pathway	0.814908	0	1
HTLV-I infection	1	Human Diseases	ko05166	HTLV-I infection	0.852999	0	1
Pathways in cancer	1	Human Diseases	ko05200	Pathways in cancer	0.953481	0	1
Neuroactive ligand-receptor interaction	5	Environmental Information Processing	ko04080	Neuroactive ligand-receptor interaction	0.013525	5	0
Influenza A	5	Human Diseases	ko05164	Influenza A	0.018855	5	0
Lysosome	5	Cellular Processes	ko04142	Lysosome	0.041495	5	0
Steroid biosynthesis	4	Metabolism	ko00100	Steroid biosynthesis	0.003277	4	0
Cholesterol metabolism	4	Organismal Systems	ko04979	Cholesterol metabolism	0.013444	4	0
Ubiquinone and other terpenoid-quinone biosynthesis	2	Metabolism	ko00130	Ubiquinone and other terpenoid-quinone biosynthesis	0.092699	2	0
Signaling pathways	2	Cellular Processes	ko04550	Signaling pathways regulating	0.17208	2	0

regulating pluripotency of stem cells				pluripotency of stem cells			
Toll and Imd signaling pathway	2	Organismal Systems	ko04624	Toll and Imd signaling pathway	0.17706	2	0
Drug metabolism - other enzymes	2	Metabolism	ko00983	Drug metabolism - other enzymes	0.310707	2	0
Parkinson disease	2	Human Diseases	ko05012	Parkinson disease	0.371895	2	0
Maturity onset diabetes of the young	1	Human Diseases	ko04950	Maturity onset diabetes of the young	0.105915	1	0
Cytokine-cytokine receptor interaction	1	Environmental Information Processing	ko04060	Cytokine-cytokine receptor interaction	0.189483	1	0
Galactose metabolism	1	Metabolism	ko00052	Galactose metabolism	0.233735	1	0
Starch and sucrose metabolism	1	Metabolism	ko00500	Starch and sucrose metabolism	0.254961	1	0
Pentose phosphate pathway	1	Metabolism	ko00030	Pentose phosphate pathway	0.295693	1	0
ECM-receptor interaction	1	Environmental Information Processing	ko04512	ECM-receptor interaction	0.31523	1	0
Arrhythmogenic right ventricular cardiomyopathy (ARVC)	1	Human Diseases	ko05412	Arrhythmogenic right ventricular cardiomyopathy (ARVC)	0.324797	1	0
Glyoxylate and	1	Metabolism	ko00630	Glyoxylate and dicarboxylate	0.343539	1	0

dicarboxylate metabolism				metabolism			
Cocaine addiction	1	Human Diseases	ko05030	Cocaine addiction	0.352717	1	0
Steroid hormone biosynthesis	1	Metabolism	ko00140	Steroid hormone biosynthesis	0.388184	1	0
Pentose and glucuronate interconversions	1	Metabolism	ko00040	Pentose and glucuronate interconversions	0.437829	1	0
Serotonergic synapse	1	Organismal Systems	ko04726	Serotonergic synapse	0.437829	1	0
Chemical carcinogenesis	1	Human Diseases	ko05204	Chemical carcinogenesis	0.437829	1	0
Synaptic vesicle cycle	1	Organismal Systems	ko04721	Synaptic vesicle cycle	0.461144	1	0
Cardiac muscle contraction	1	Organismal Systems	ko04260	Cardiac muscle contraction	0.468702	1	0
Insulin secretion	1	Organismal Systems	ko04911	Insulin secretion	0.468702	1	0
Drug metabolism - cytochrome P450	1	Metabolism	ko00982	Drug metabolism - cytochrome P450	0.476156	1	0
Metabolism of xenobiotics by cytochrome P450	1	Metabolism	ko00980	Metabolism of xenobiotics by cytochrome P450	0.490756	1	0
Amphetamine addiction	1	Human Diseases	ko05031	Amphetamine addiction	0.490756	1	0
Porphyrin and chlorophyll metabolism	1	Metabolism	ko00860	Porphyrin and chlorophyll metabolism	0.511909	1	0
Glycerophospholipid metabolism	1	Metabolism	ko00564	Glycerophospholipid metabolism	0.576366	1	0
Retinol metabolism	1	Metabolism	ko00830	Retinol metabolism	0.582332	1	0
Inflammatory mediator	1	Organismal Systems	ko04750	Inflammatory mediator regulation	0.588214	1	0

regulation of TRP channels				of TRP channels			
Retrograde endocannabinoid signaling	1	Organismal Systems	ko04723	Retrograde endocannabinoid signaling	0.605377	1	0
Adrenergic signaling in cardiomyocytes	1	Organismal Systems	ko04261	Adrenergic signaling in cardiomyocytes	0.632432	1	0
Dopaminergic synapse	1	Organismal Systems	ko04728	Dopaminergic synapse	0.632432	1	0
Alcoholism	1	Human Diseases	ko05034	Alcoholism	0.642736	1	0
Oxytocin signaling pathway	1	Organismal Systems	ko04921	Oxytocin signaling pathway	0.647781	1	0
Glutathione metabolism	1	Metabolism	ko00480	Glutathione metabolism	0.67661	1	0
Non-alcoholic fatty liver disease (NAFLD)	1	Human Diseases	ko04932	Non-alcoholic fatty liver disease (NAFLD)	0.742621	1	0
mTOR signaling pathway	1	Environmental Information Processing	ko04150	mTOR signaling pathway	0.75343	1	0
Proteoglycans in cancer	1	Human Diseases	ko05205	Proteoglycans in cancer	0.76715	1	0
MAPK signaling pathway	1	Environmental Information Processing	ko04010	MAPK signaling pathway	0.792375	1	0
Peroxisome	1	Cellular Processes	ko04146	Peroxisome	0.83739	1	0
Thermogenesis	1	Organismal Systems	ko04714	Thermogenesis	0.889921	1	0
Alzheimer disease	1	Human Diseases	ko05010	Alzheimer disease	0.937623	1	0
Huntington disease	1	Human Diseases	ko05016	Huntington disease	0.9437	1	0

Table S9. List of gene ontology (GO) terms along with up and down-regulated genes

GO ID (level1)	GO Term (level1)	GO ID (level2)	GO Term (level2)	number_of_Ind-C-vs-Ind-30 (up)	number_of_Ind-C-vs-Ind-30 (down)
GO:0008150	Biological Process	GO:0008152	metabolic process	33	18
GO:0008150	Biological Process	GO:0022610	biological adhesion	2	0
GO:0008150	Biological Process	GO:0002376	immune system process	1	0
GO:0008150	Biological Process	GO:0051179	localization	9	2
GO:0008150	Biological Process	GO:0032502	developmental process	1	0
GO:0008150	Biological Process	GO:0023052	signaling	4	1
GO:0008150	Biological Process	GO:0050896	response to stimulus	5	1
GO:0008150	Biological Process	GO:0050789	regulation of biological process	8	2
GO:0008150	Biological Process	GO:0065007	biological regulation	8	2
GO:0008150	Biological Process	GO:0032501	multicellular organismal process	2	0
GO:0008150	Biological Process	GO:0071840	cellular component organization or biogenesis	1	0
GO:0008150	Biological Process	GO:0009987	cellular process	16	14
GO:0003674	Molecular Function	GO:0003824	catalytic activity	30	19
GO:0003674	Molecular Function	GO:0098772	molecular function regulator	3	1
GO:0003674	Molecular Function	GO:0005215	transporter activity	6	1
GO:0003674	Molecular Function	GO:0140110	transcription regulator activity	1	1
GO:0003674	Molecular Function	GO:0005488	binding	30	20
GO:0003674	Molecular Function	GO:0060089	molecular transducer activity	1	0
GO:0005575	Cellular Component	GO:0044425	membrane part	12	3
GO:0005575	Cellular Component	GO:0016020	membrane	16	5
GO:0005575	Cellular Component	GO:0005576	extracellular region	3	0
GO:0005575	Cellular Component	GO:0043226	organelle	3	2
GO:0005575	Cellular Component	GO:0044422	organelle part	0	1
GO:0005575	Cellular Component	GO:0044464	cell part	4	4
GO:0005575	Cellular Component	GO:0005623	cell	4	4
GO:0005575	Cellular Component	GO:0032991	protein-containing complex	1	1
